# Supplementary material for: Leucine Zipper-Bearing Kinase Is a Critical Regulator of Astrocyte Reactivity in the Adult Mammalian CNS
Source: Cell Rep. Author manuscript; Available in PMC 2018 Apr 18. (PMC5905706; doi:10.1016/j.celrep.2018.02.102)
Supplement: 1 [file NIHMS957794-supplement-1.pdf]

Cell Reports, Volume 22

Supplemental Information

Leucine Zipper-Bearing Kinase

Is a Critical Regulator of Astrocyte Reactivity

in the Adult Mammalian CNS

Meifan Chen, Cédric G. Geoffroy, Jessica M. Meves, Aarti Narang, Yunbo Li, Mallorie T. Nguyen, Vung S. Khai, Xiangmei Kong, Christopher L. Steinke, Krislyn I. Carolino, Lucie Elzière, Mark P. Goldberg, Yishi Jin, and Binhai Zheng

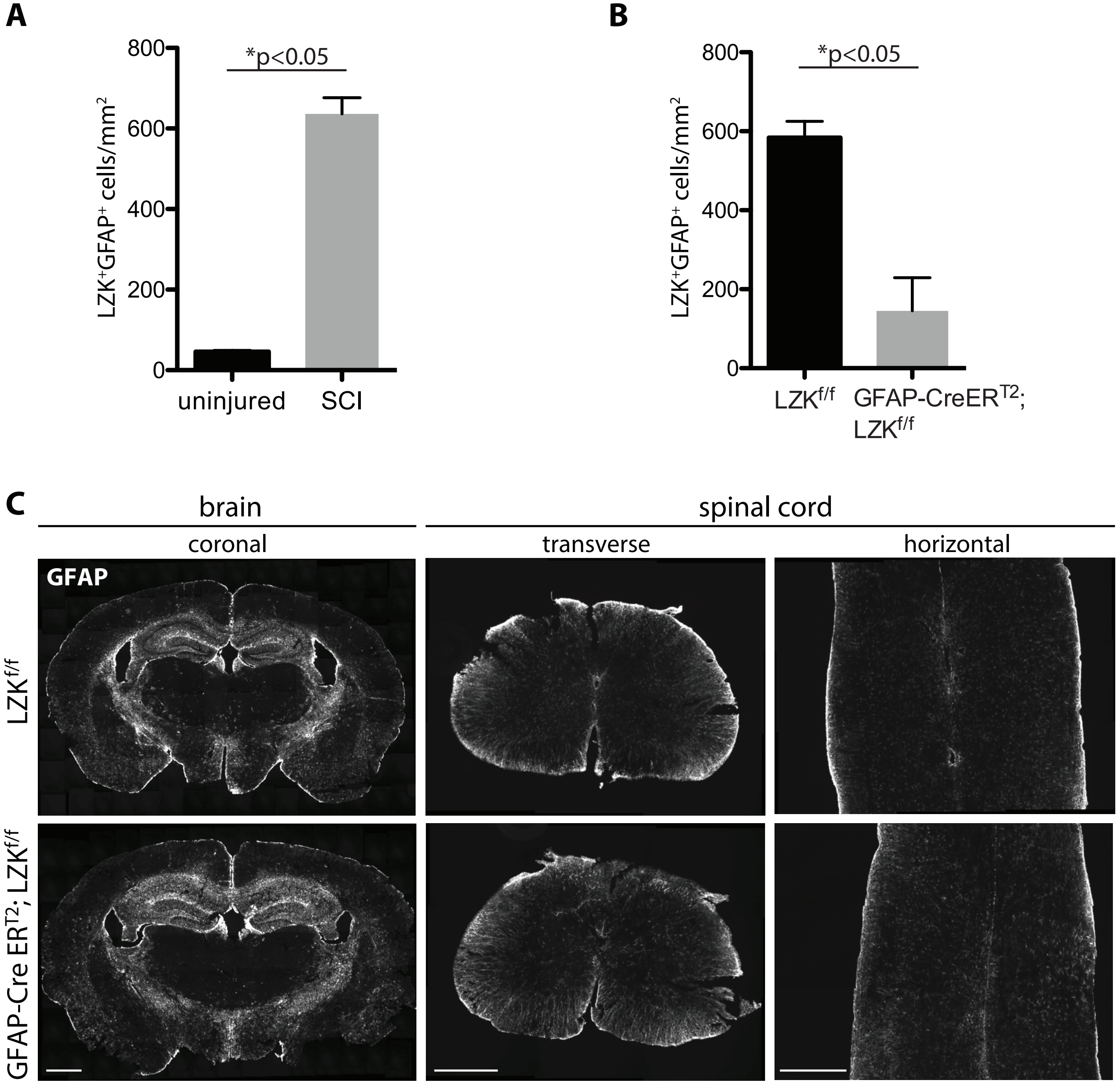

Figure S1. LZK deletion in adult astrocytes diminished injury-induced LZK upregulation and had no effect on GFAP expression in the uninjured CNS. Related to Figure 1. (A) Quantification of LZK^+GFAP^+ cell number in the uninjured spinal cords as compared to injured spinal cords of wildtype mice at 14 days post injury (dpi), perilesional area 0.5-1mm from the injury site. N=3 per condition, *$p<0.05$ by unpaired parametric t-test. Error bar, SEM. (B) Quantification of LZK^+GFAP^+ cell number in the injured spinal cords of control $LZK^{f/f}$ mice as compared to that of GFAP-$CreER^{T2}$;$LZK^{f/f}$ mice at 14 dpi, perilesional area 0.5-1mm from the injury site. N=3 per genotype, *$p<0.05$ by unpaired parametric t-test. Error bar, SEM. (C) Immunofluorescence staining of GFAP on coronal sections of the brain, and transverse and horizontal sections of the spinal cord from tamoxifen-treated $LZK^{f/f}$ control and GFAP-$CreER^{T2}$;$LZK^{f/f}$ mice. Scale bar = 1 mm (brain), 500 µm (spinal cord). Figures are composites of smaller microscopy images.

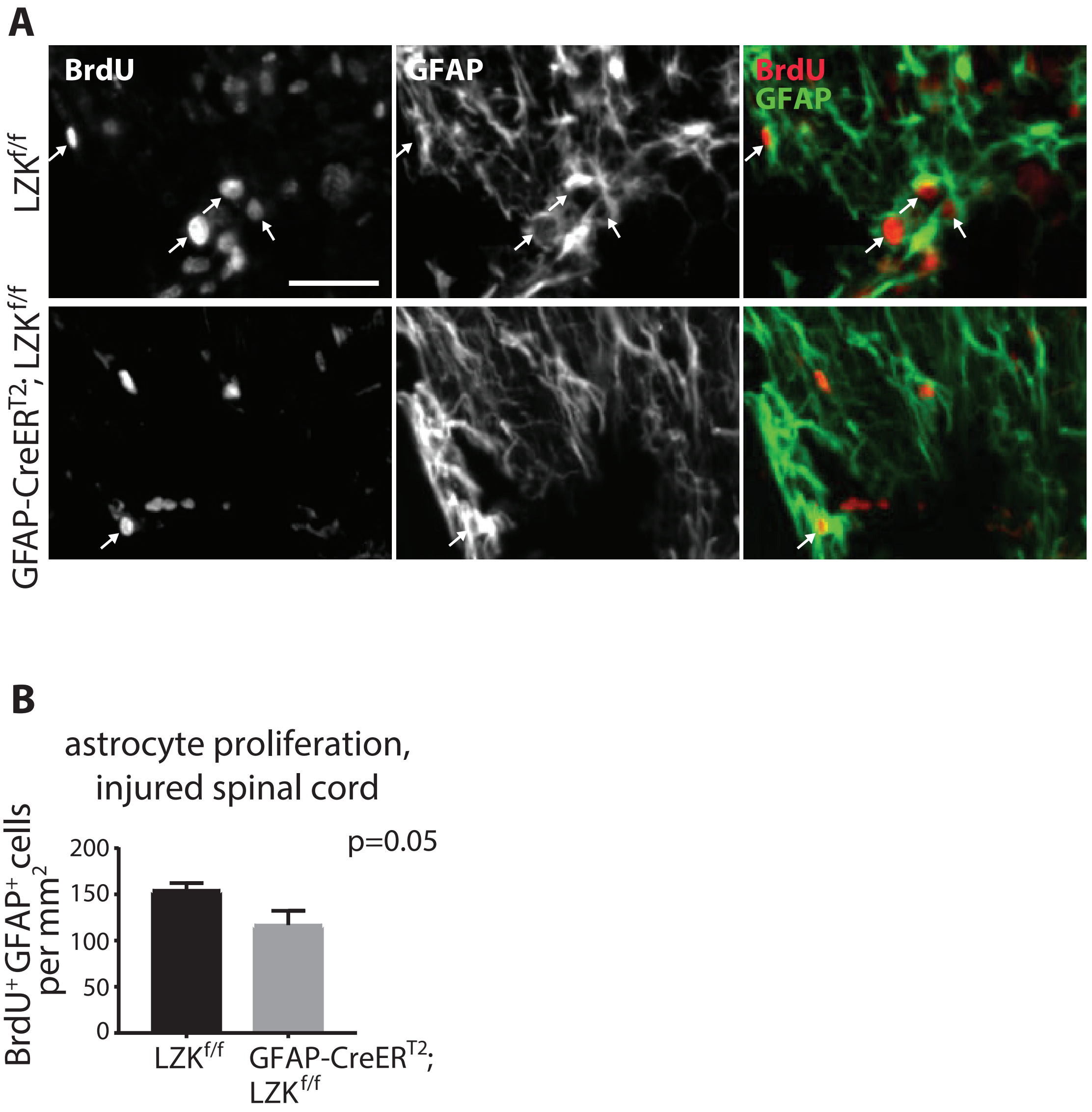

Figure S2. Reduced astrocyte proliferation in injured mice depleted of astrocytic LZK. Related to Figure 3. (A) Representative images of BrdU and GFAP co-immunofluorescence staining within 250 µm of spinal cord injury site of tamoxifen-treated LZK$^{f/f}$ control and GFAP-CreER^{T2};LZK$^{f/f}$ mice sacrificed on 14 dpi. Scale bar = 50 µm. The number of BrdU+ nuclei tightly encased by or overlapping with GFAP+ astrocytic processes (arrows) is quantified in (B). Control, N=5; astrocytic LZK knockout mice, N=3. *p*=0.05 by two-tailed unpaired parametric t-test. Error bar, SEM.

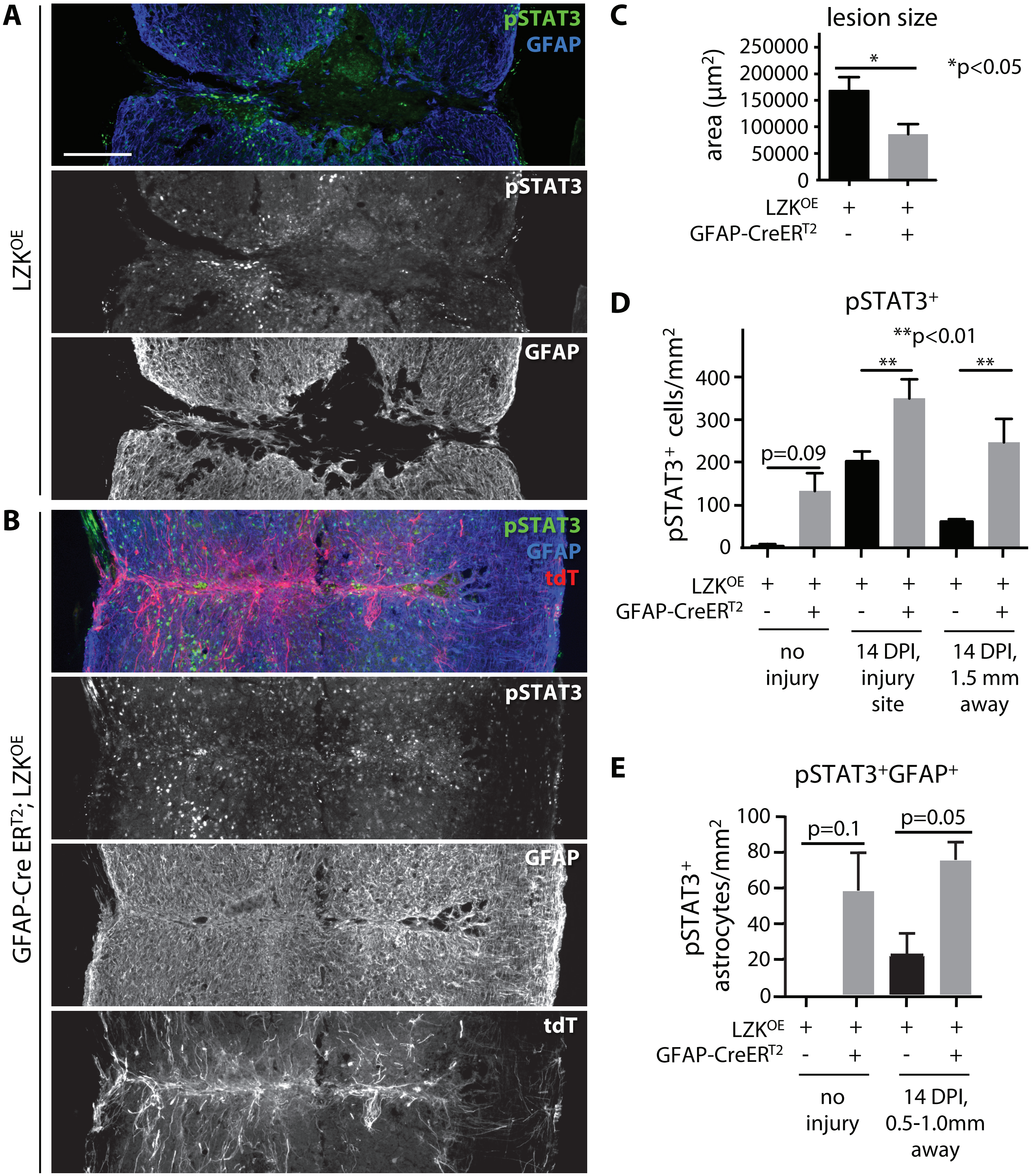
A
pSTAT3
GFAP
pSTAT3
GFAP
LZKOE
B
pSTAT3
GFAP
tdT
pSTAT3
GFAP
tdT
GFAP-Cre ERT2; LZKOE
C
lesion size
area (μm2)
250000
200000
150000
100000
50000
0
*
*p<0.05
LZKOE + +
GFAP-CreERT2 - +
D
pSTAT3+
pSTAT3+ cells/mm2
**p<0.01
400
300
200
100
0
p=0.09
**
**
LZKOE + + + + + +
GFAP-CreERT2 - + - + - +
no injury
14 DPI, injury site
14 DPI, 1.5 mm away
E
pSTAT3+GFAP+
pSTAT3+ astrocytes/mm2
100
80
60
40
20
0
p=0.1
p=0.05
LZKOE + + + +
GFAP-CreERT2 - + - +
no injury
14 DPI, 0.5-1.0mm away

Figure S3

Figure S3. Confirmation of reduced injury size in mice overexpressing LZK in astrocytes in an independent surgical experiment and assessment of STAT3 activation in astrocytes in the injured spinal cord. Related to Figure 4. (A,B) Immunofluorescence detection of pSTAT3, GFAP and tdT on horizontal spinal cord sections of LZK^{OE} control (A) and GFAP-$CreER^{T2}$; LZK^{OE} mice (B) 14 days after spinal cord injury. This injury experiment was done by a different surgeon as that shown in Fig. 3. Note the elongated morphologies of tdT^{+} cells and processes lining up at the injury site in GFAP-$CreER^{T2}$; LZK^{OE} mice. Scale bar = 200 µm. (C) Quantification of the lesion area 14 days post injury. (D) Quantification of the number of $pSTAT3^{+}$ cells in uninjured mice and 14 days post injury (at the injury site and 1.5 mm away from the lesion site). Note that LZK overexpression in astrocytes increased $pSTAT3^{+}$ cells in the spinal cord of both uninjured and injured mice as compared with non-overexpression controls. (E) Quantification of the number of $pSTAT3^{+}GFAP^{+}$ cells in uninjured mice and 14 days post injury (at the injury site and 1.5 mm away from the lesion site). Note that LZK overexpression in astrocytes increased the number of astrocytes with activated STAT3 (pSTAT3) in the spinal cord of both uninjured and injured mice as compared with non-overexpression controls. N = 2 per genotype (no injury); 7 (LZK^{OE} mice, 14 days post injury), 5 (GFAP-$CreER^{T2}$;LZK^{OE}, 14 days post injury), *p<0.05, **p<0.01 by unpaired parametric t-test. Error bar, SEM. Figures are composites of smaller microscopy images.

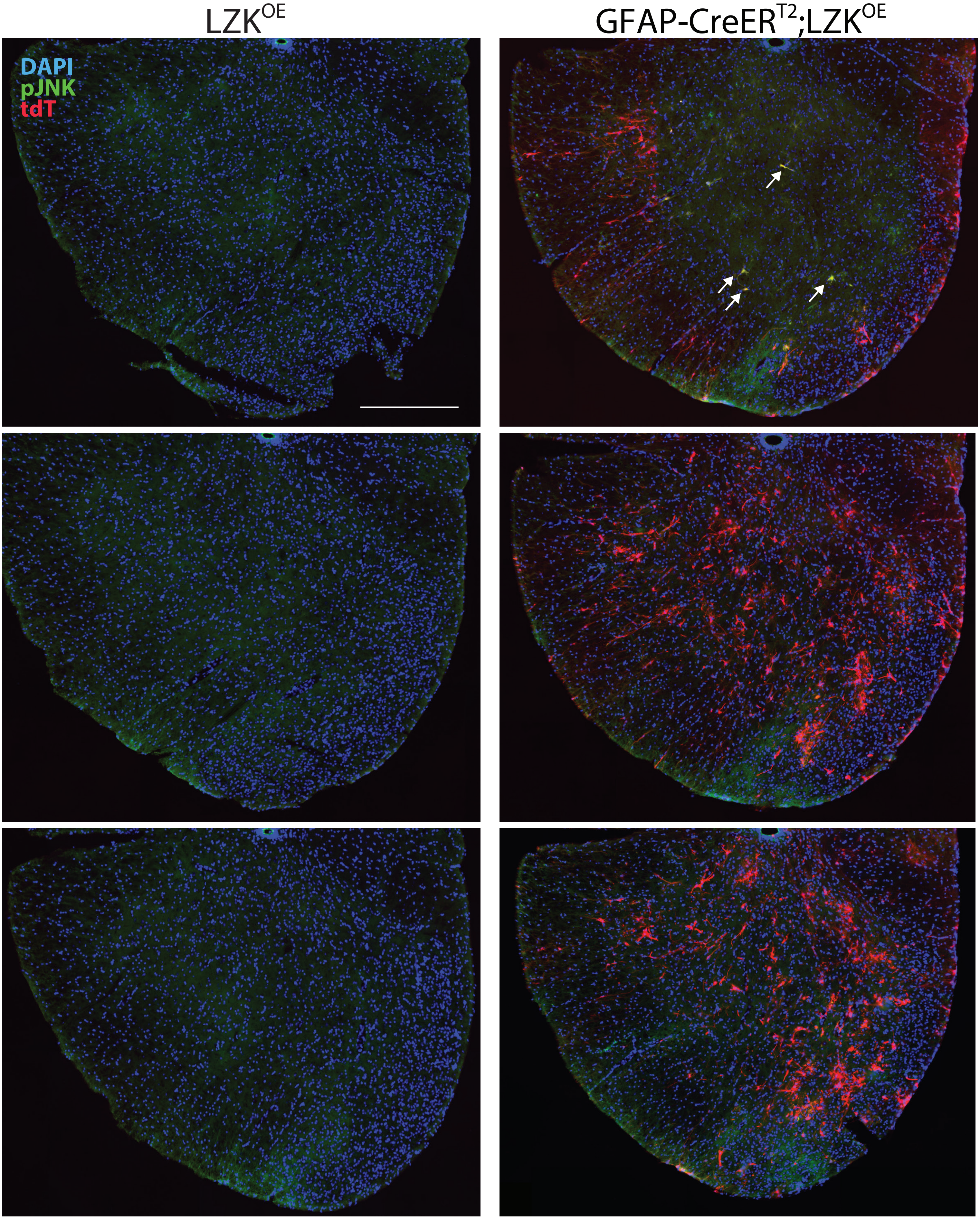

Figure S4

Figure S4. Infrequent detection of JNK activation in adult astrocytes overexpressing LZK without injury. Related to Figure 5. Representative images of DAPI, pJNK, and tdTomato (tdT) immunofluorescence co-labeling in uninjured spinal cords of control LZK^{OE} and $GFAP\text{-}CreER^{T2}$;LZK^{OE} mice 3 weeks after the last tamoxifen treatment. Representative sections from 3 control and 3 astrocytic LZK-overexpressing mice are shown (one image per mouse). JNK activation (as assessed by pJNK immunoreactivity) was observed in astrocytes overexpressing LZK-tdT in only one $GFAP\text{-}CreER^{T2}$; LZK^{OE} mouse (arrows). Scale bar = 500 µm. Figures are composites of smaller images.

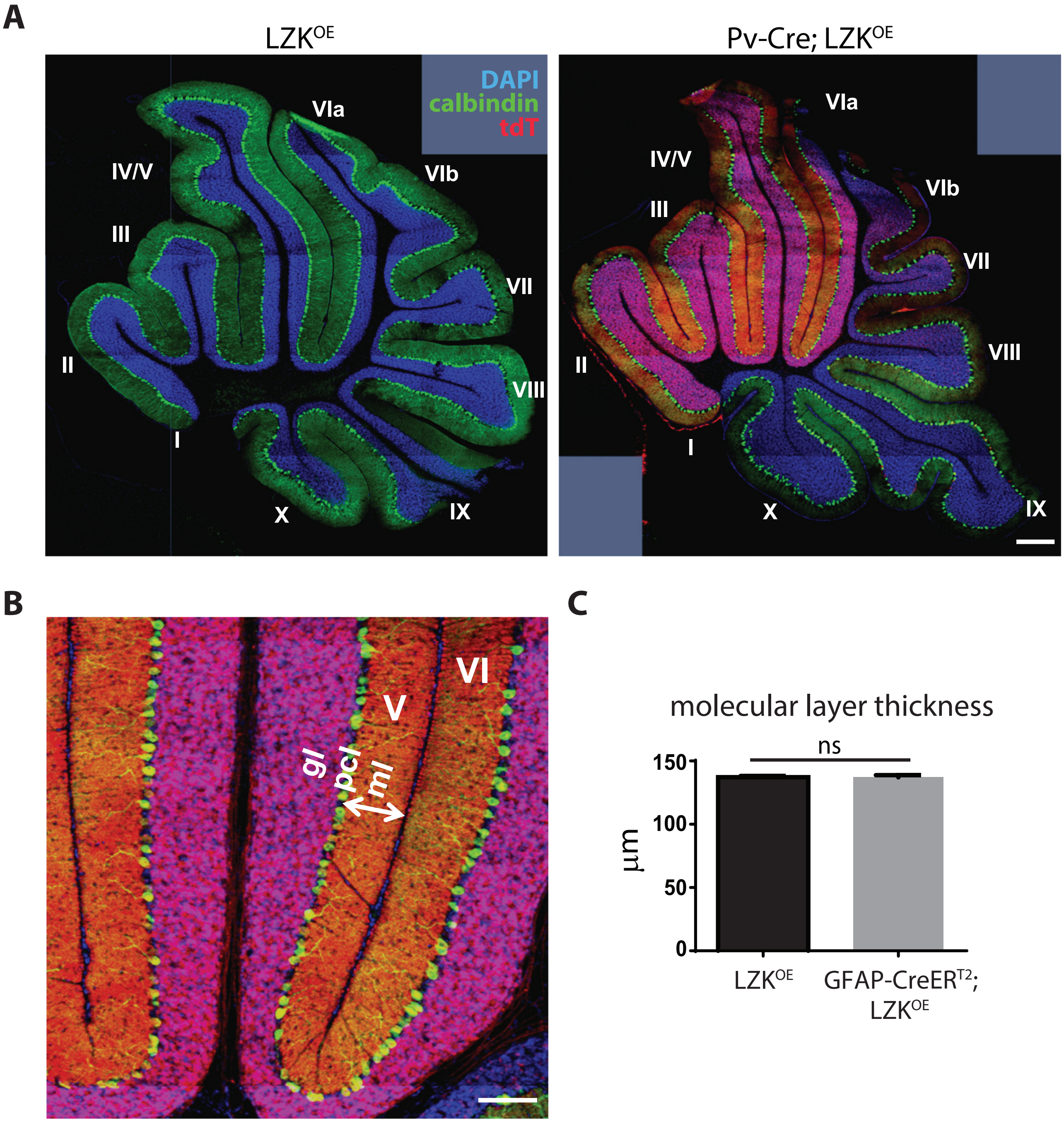

Figure S5. Absence of non-physiological effects of LZK overexpression in parvalbumin (Pv)-expressing neurons. Related to Figure 5. (A) Representative mid-sagittal cerebellar sections from control LZK^{OE} and Pv-Cre;LZK^{OE} mice, at post-natal day 21, co-labeled with Calbindin (Purkinje cell marker), DAPI, and tdTomato. Cerebellar lobules are labeled from I-X. Scale bars = 300 µm. Transgene expression is stronger in Lobules I-V of the cerebellum anterior lobe and lobule VIa of the posterior lobe compared to the other lobules (Hippenmeyer et al, 2005), as shown by tdTomato expression. (B) Representative image showing how molecular layer thickness is measured within the primary fissure. Scale bar = 100 µm. (C) Quantification of molecular layer thickness. Molecular layer (ml); Purkinje cell layer (pcl); granular layer (gl); ns, not significant. Error bar, SEM. Figures are composites of smaller microscopy images.

SUPPLEMENTAL EXPERIMENTAL PROCEDURES

BrdU injections. BrdU (Sigma, B5002) treatment was injected intraperitoneally at 100mg/kg (in 0.007N NaOH and saline) once daily on days 2 through 5 after spinal cord injury.

General histology. Terminal anesthesia was carried out by intraperitoneal injection of pentobarbital sodium or isoflurane overdose. Mice were perfused transcardially with 4% paraformaldehyde, in which dissected brains and spinal cords were further post-fixed overnight at 4°C. Tissues were then cryoprotected in 30% sucrose overnight at 4°C and embedded in O.C.T. compound (Fisher HealthCare) on dry ice. Embedded tissues were sectioned at 20 µm thickness by cryostat (Leica), collected in PBS with 0.01% sodium azide, and further processed for histological examination (see below for details on staining, antibodies and quantification).

Fluorescent immunohistochemistry. Tissue sections were stained as free floating sections. They were first washed twice (wash buffer: 0.2% triton, 1X PBS), at 10 min each. They were then blocked and permeabilized (0.4% triton, 5% sera matching the species of the secondary antibodies, 1X PBS) for 1 hr at room temperature. Incubation in antibody solution (0.2% triton, 1% matching sera, 0.01% sodium azide, 1X PBS) containing desired primary antibody (see concentrations below) was carried out at room temperature overnight. Sections were washed three times, 10 min each, followed by secondary antibody staining for 2 hr at room temperature (antibody solution with desired secondary antibodies used at 1:500). Sections were washed three times, incubated with DAPI (1 µg/ml, 1X PBS) for 10 min, followed by mounting onto glass slides (Fisher Scientific) and cover slipping (Fisher Scientific) with Fluoromount-G (Southern Biotech). For BrdU staining, spinal cords were first incubated in 2N HCl at 37°C for 30 min, rinsed in 0.1M borate buffer at room temperature twice (10 min each), then washed in PBS twice (10 min each). Sections were then subjected to general staining protocol described above with anti-BrdU and anti-GFAP antibodies. For pSTAT3 staining that requires antigen retrieval, spinal cord sections were pre-treated with 1% NaOH for 20 min at room temperature, followed by 3 washes with PBS (10 min each), then incubated with 0.3% glycine in PBS for 10 min, rinsed with PBS (3 times, 10 min each), and finally treated with 0.03% sodium dodecyl sulfate (SDS) in PBS for 10 minutes. After 3 additional washes, sections were blocked in 5% Normal Horse Serum (NHS) in 0.2% Triton X-100 in PBS (PBS-TX) for 1 hr at room temperature, and then incubated with anti-pSTAT3 antibody for overnight at room temperature. The next day, sections were washed 3 times, 10 min per wash in PBS-TX and then incubated in biotinylated anti-rabbit (1:250, in PBS) for 2 hr at room temperature. After 3 washes (10 min each), sections were incubated with ABC solution (in 0.1% Tween-20 1X PBS, Vector Laboratories) over night at 4°C. On the third day, sections were washed with PBS (4 times, 30 min each) then with TSA (Alexa Fluor 488, 1:200 in PBS, Perkin Elmer) for 10 min. After 3 additional washes in PBS, sections were stained for GFAP and tdTomato as described above.

Antibodies. Commercially available antibodies used in this study were: LZK (1:500, rabbit, R06696; Sigma-Aldrich), vimentin (1:500, chicken, ab24525, Abcam), SOX9 (1:500, goat, AF3075, R&D Systems), Ki67 (1:500, rabbit, RM-9106, Thermo Fisher Scientific), GFAP (1:500, rabbit, Z0334, Dako), GFAP (1:500, rat, 130300, Life Technologies), pSTAT3-Tyr705 (1:100, rabbit, 9145, Cell Signaling), tdTomato (1:500, goat, AB8181-200, SICGEN), phospho-JNK(Thr183/Tyr185) (1:300, rabbit, 4668, Cell Signaling), calbindin (1:500, rabbit, 13176, Cell Signaling), BrdU (1:500, rat, ab6326, abcam), Alexa Fluor-tagged secondary antibodies used were Alexa 488, Alexa 546 and Alexa 647 (Thermo Fisher Scientific).

Microscopy and quantification. Stained tissue sections were photographed using an upright epifluorescence microscopy (Zeiss Axio Scan.Z1 and Zeiss Axio Imager M1). After image acquisition, immunofluorescence signal intensity, lesion size, and cell count were determined using the image analysis software ImageJ. For analyses on uninjured brain and spinal cord, 3 mice per genotype, and 3 sections per mouse comparable across animals were used. GFAP immunofluorescence intensity in these animals was quantified as follows: in each coronal brain section, four sampling frames (each of area 440,000 μm^2) were placed within the region of cerebral cortex. In the spinal cord, nine sampling frames or zones (each of length 250 µm and covering the entire width of the cord) were used per section. For SOX9 immunofluorescence intensity quantification, 50 sampling frames (each of area 87 μm^2 surrounding a single $SOX9^+$ nucleus) were placed within the region of interest per section (either the cerebral cortex or gray matter of the spinal cord). For evaluation of signal intensity, integrated density was averaged after subtraction of background signal, and unpaired parametric t-test was used to calculate statistical significance in difference between two groups (GraphPad Prism software). For cell counts, 3 sampling frames of known area were placed within the region of interest per section. All cells positive for signal of interest within each frame was counted (200-600 cells per frame) and normalized to area. Unpaired parametric t-test was used for statistical

evaluation between two groups. To quantify GFAP immunofluorescence intensity in injured spinal cords, 2-3 sections containing the lesion site spanning the entire width of the cord were examined per mouse (see figure legends for group size of spinal cord injury experiments). Nine zones as described above were placed on each section, with the first on the lesion border and the rest placed sequentially away from the injury site and immediately adjacent to each other. After subtraction of background signal, average integrated density for each zone was individually calculated; multiple t-test (paired analysis per zone) was used for statistical evaluation. Vimentin intensity in the injured spinal cord was measured similarly, with only the first zone within 250 µm of the lesion border applied for quantification. Spinal cord injury size was quantified by tracing $GFAP^+$ lesion border in all lesion-containing sections in a set of step-serial horizontal sections (section thickness of 20 µm, staining every 6th section). Lesion area was averaged and unpaired parametric t-test was used to assess statistical significance between two groups. For $pSTAT3^+$ cell counts, the total number of pSTAT3/DAPI co-stained nuclei was quantified at 2 different distances (0-500 µm from the injury site and 1.5-2.0 mm away from the injury) and normalized to the surface of the section measured. 2 sections containing the lesion site spanning the entire width of the cord were examined per mouse (see figure legends for group size). Unpaired parametric t-test was used for statistical evaluation between two groups. For $pSTAT3^+GFAP^+$ cell counts, the number of $pSTAT3^+$ cells overlapping with or tightly encased by $GFAP^+$ astrocytic processes were quantified within a sample region of 200 µm radius. Number of cells were normalized to the area quantified. Two histological sections per mouse were used to generate average cell count per mouse. N = 2 per genotype with no injury; N = 3 per genotype with injury. Unpaired parametric t-test was used for statistical evaluation between two genotypes per condition. For astrocyte proliferation quantification, either the total number of $Ki67^+SOX9^+$ co-labeled nuclei, or the total number of $BrdU^+$ nuclei tightly encased by/overlapping with $GFAP^+$ astrocytic processes within a region spanning the entire width of the spinal cord and within 250 µm of the lesion border (excluding ependymal cells lining the central canal) was counted and normalized to area. Two sections per mouse were quantified. Unpaired parametric t-test was used for statistical evaluation between two genotypes. Thickness of the cerebellar molecular layer was measured within the primary fissure between lobules V and VI on sagittal sections. 2-3 midline sections per mouse, three mice per genotype were used for quantification. Unpaired t-test was used for statistical evaluation between two genotypes. To quantify the number of LZK^+GFAP^+ cells in the uninjured and injured spinal cords, such cells in a sample region of grey matter approximately $0.2mm^2$ in size were counted and normalized to the size of the area quantified. Two histological sections per animal were used to generate average cell count per animal. N = 3 per condition/genotype. Unpaired parametric t-test was used for statistical evaluation between two groups.
