# Supplementary material for: Leucine Zipper-Bearing Kinase Is a Critical Regulator of Astrocyte Reactivity in the Adult Mammalian CNS
Source: Cell Rep. Author manuscript; Available in PMC 2018 Apr 18. (PMC5905706; doi:10.1016/j.celrep.2018.02.102)
Supplement: 2 [file NIHMS957794-supplement-2.pdf]

Cell Reports

Article

Leucine Zipper-Bearing Kinase Is a Critical Regulator of Astrocyte Reactivity in the Adult Mammalian CNS

Graphical Abstract

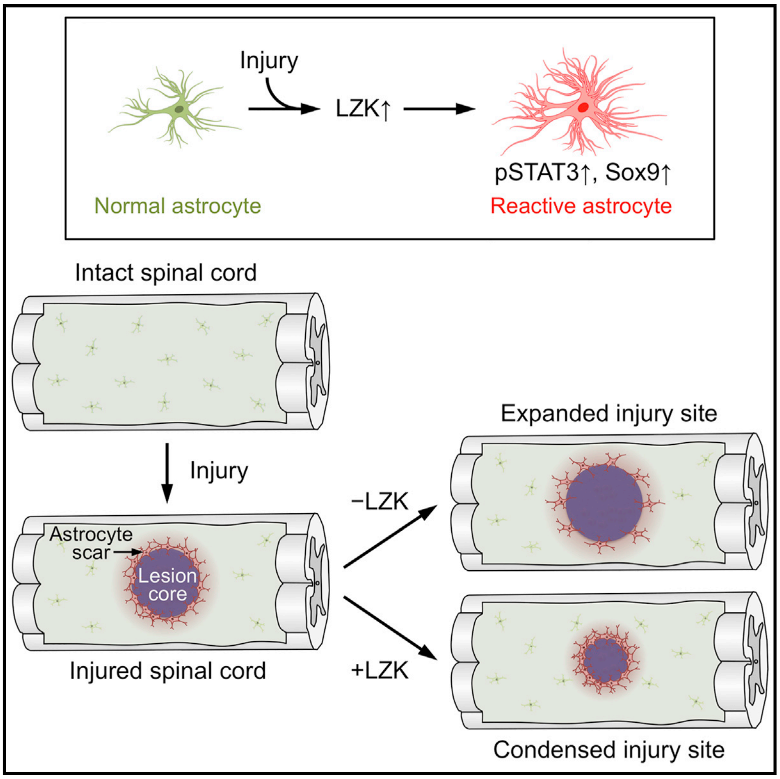

Highlights

- LZK (MAP3K13) is upregulated in astrocytes after spinal cord injury in adult mice
- Astrocytic LZK deletion in adult mice reduces astrogliosis and impairs scar formation
- Astrocytic LZK overexpression in adult mice enhances astrogliosis and scar formation
- Astrocytic LZK overexpression in uninjured adult CNS induces widespread astrogliosis

Authors

Meifan Chen, Cédric G. Geoffroy, Jessica M. Meves, ..., Mark P. Goldberg, Yishi Jin, Binhai Zheng

Correspondence

mark.goldberg@utsouthwestern.edu (M.P.G.),
yijin@ucsd.edu (Y.J.),
binhai@ucsd.edu (B.Z.)

In Brief

Reactive astrocytes are recognized increasingly for their role in CNS injury and disease. Chen et al. find that leucine zipper-bearing kinase (LZK) is a positive regulator of astrocyte reactivity that controls glial scar formation after spinal cord injury. These findings have broad implications for understanding injury responses and promoting neural repair.

Chen et al., 2018, Cell Reports *22*, 3587–3597
March 27, 2018 © 2018 The Author(s).
https://doi.org/10.1016/j.celrep.2018.02.102

CellPress

Cell Reports
Article

OPEN ACCESS
CellPress

Leucine Zipper-Bearing Kinase Is a Critical Regulator of Astrocyte Reactivity in the Adult Mammalian CNS

Meifan Chen,[1,2] Cédric G. Geoffroy,[1,4] Jessica M. Meves,[1] Aarti Narang,[1] Yunbo Li,[3] Mallorie T. Nguyen,[1] Vung S. Khai,[1] Xiangmei Kong,[2] Christopher L. Steinke,[3] Krislyn I. Carolino,[1] Lucie Elzière,[1] Mark P. Goldberg,[2,*] Yishi Jin,[1,3,*] and Binhai Zheng[1,5,*]
[1]Department of Neurosciences, School of Medicine, University of California San Diego, La Jolla, CA 92093, USA
[2]Department of Neurology and Neurotherapeutics, University of Texas Southwestern Medical Center, Dallas, TX 75390, USA
[3]Section of Neurobiology, Division of Biological Sciences, University of California San Diego, La Jolla, CA 92093, USA
[4]Present address: Department of Neuroscience and Experimental Therapeutics, College of Medicine, Texas A&M University Health Science Center, Bryan, TX 77807, USA
[5]Lead Contact
*Correspondence: mark.goldberg@utsouthwestern.edu (M.P.G.), yijin@ucsd.edu (Y.J.), binhai@ucsd.edu (B.Z.)
https://doi.org/10.1016/j.celrep.2018.02.102

SUMMARY

Reactive astrocytes influence post-injury recovery, repair, and pathogenesis of the mammalian CNS. Much of the regulation of astrocyte reactivity, however, remains to be understood. Using genetic loss and gain-of-function analyses *in vivo*, we show that the conserved MAP3K13 (also known as leucine zipper-bearing kinase [LZK]) promotes astrocyte reactivity and glial scar formation after CNS injury. Inducible LZK gene deletion in astrocytes of adult mice reduced astrogliosis and impaired glial scar formation, resulting in increased lesion size after spinal cord injury. Conversely, LZK overexpression in astrocytes enhanced astrogliosis and reduced lesion size. Remarkably, in the absence of injury, LZK overexpression alone induced widespread astrogliosis in the CNS and upregulated astrogliosis activators pSTAT3 and SOX9. The identification of LZK as a critical cell-intrinsic regulator of astrocyte reactivity expands our understanding of the multicellular response to CNS injury and disease, with broad translational implications for neural repair.

INTRODUCTION

Astrocytes have diverse functions in the healthy CNS and participate in CNS pathophysiology. In the healthy CNS, astrocytes provide structural and metabolic support, regulate neurotransmitter uptake and synaptic transmission, and help maintain the blood-brain barrier (Ben Haim and Rowitch, 2017; Khakh and Sofroniew, 2015). Under pathological conditions, astrocytes become reactive, undergoing a spectrum of phenotypic changes from upregulation of molecular markers and cytoskeletal hypertrophy to cell proliferation (Gallo and Deneen, 2014; Liddelow and Barres, 2017). Reactive astrogliosis (or simply astrogliosis), a collective term for astrocytic responses to insults, is a common feature across a wide range of neurological conditions, including traumatic injury, stroke, epilepsy, and neurodegenerative diseases (Liddelow et al., 2017; Pekny et al., 2016). Understanding the role of astrogliosis and its molecular regulation will aid in the design of therapeutic intervention to promote recovery and repair following CNS injury and disease.

Following traumatic injury to the CNS such as a spinal cord injury, astrocytes display a gradient of responses centered around the lesion site (Burda and Sofroniew, 2014). Reactive astrocytes form a dense scar just outside the fibrotic lesion core, which consists of mainly non-neural cells such as fibroblast-like cells and macrophages. The role of reactive astrocytes and the glial scar in recovery and repair after CNS injury is complex. It is generally thought that the glial scar presents a physical barrier and produces chondroitin sulfate proteoglycans (CSPGs) that inhibit axon growth and regeneration (Bradbury et al., 2002; Silver and Miller, 2004). However, akin to wound repair following skin lesions, reactive astrocytes and the glial scar also have beneficial roles in confining the injury site, repairing the blood-brain barrier, and limiting the spread of inflammation (Bush et al., 1999; Faulkner et al., 2004; Herrmann et al., 2008; Sabelström et al., 2013; Wanner et al., 2013). Recent evidence indicates that, contrary to common belief, the astrocyte scar may even aid in axon regeneration (Anderson et al., 2016).

Despite the increasingly recognized importance of reactive astrogliosis in the pathogenesis and outcome of neurological conditions, our understanding of its molecular regulation remains limited, especially regarding cell-intrinsic mechanisms. We have previously identified leucine zipper-bearing kinase (LZK) (MAP3K13), a conserved mitogen-activated protein kinase kinase kinase (MAPKKK) upstream of c-Jun N-terminal kinase (JNK) in the MAPK pathway, as a neuron-intrinsic promoter of axonal growth in cultured CNS neurons (Chen et al., 2016). Here, we investigated the *in vivo* role of LZK after CNS injury. We found that spinal cord injury induces LZK expression prominently in astrocytes. Genetic gain and loss-of-function analyses in mice indicated that LZK is an important positive regulator of astrocyte reactivity and postinjury glial scar formation. Our study opens new avenues to manipulate astrogliosis and

This is an open access article under the CC BY license (http://creativecommons.org/licenses/by/4.0/).

OPEN ACCESS
CellPress

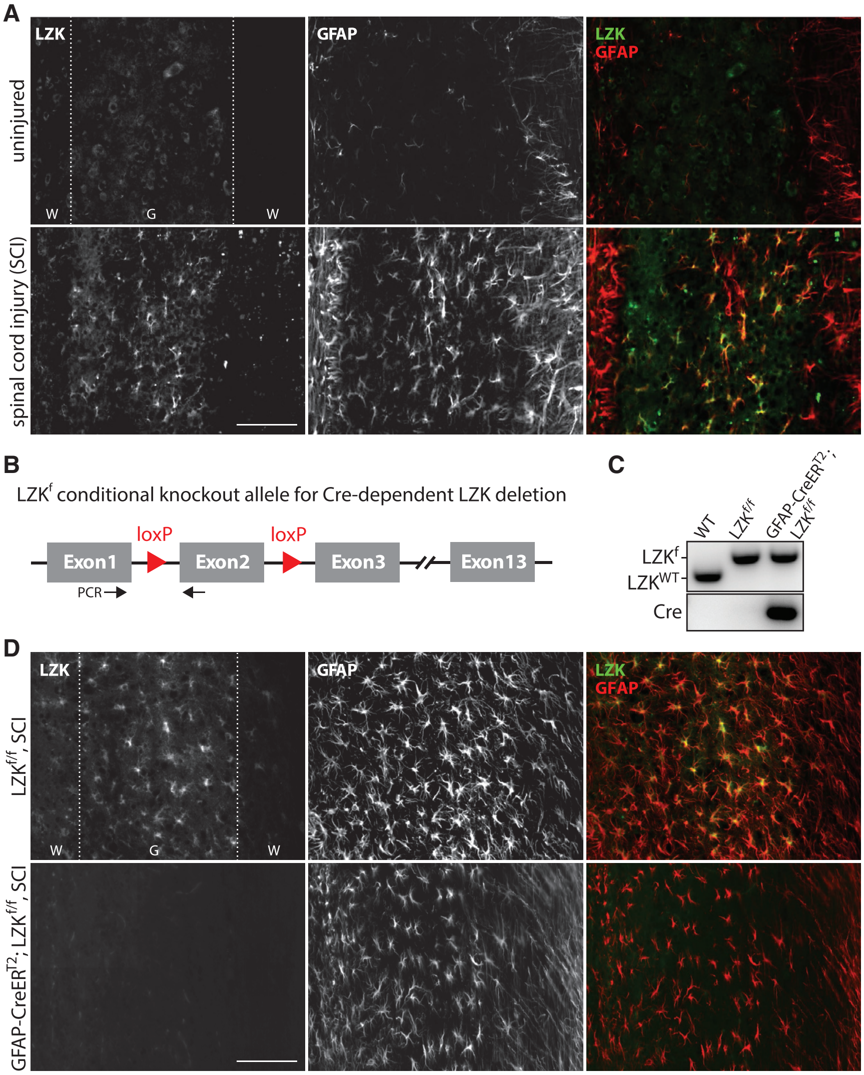

Figure 1. Injury-Induced Leucine Zipper-Bearing Kinase Expression in Astrocytes and Conditional Gene Deletion

(A) Representative images of endogenous LZK and glial fibrillary astrocyte protein (GFAP) immunostaining in the spinal cords of uninjured or injured wild-type (WT) mice (14 days after spinal cord injury [SCI]), taken 0.5–1 mm from the injury site on horizontal sections.

(B) Diagram of the LZK conditional knockout allele (LZK^{f}). Cre-mediated excision of exon 2 would result in a frameshift and thus a null allele. Black arrows mark the positions of PCR primers for genotyping.

(C) Genomic PCR genotyping of WT, LZK$^{f/f}$, and GFAP-CreER^{T2};LZK$^{f/f}$ mice.

(D) Immunostaining of endogenous LZK and GFAP in the spinal cords of tamoxifen-pretreated LZK$^{f/f}$ control and GFAP-CreER^{T2};LZK$^{f/f}$ mice 14 days after spinal cord injury (SCI), with images taken 0.5–1 mm from the injury site. Dotted lines demarcate white (W) matter and gray (G) matter. Scale bars represent 100 μm. See also Figure S1.

to understand its complex roles in the pathogenesis of and recovery from CNS injury and disease.

RESULTS

Spinal Cord Injury Upregulates LZK Expression in Astrocytes

LZK is normally expressed in the CNS of embryonic and adult mice based on mouse transcriptomic data (Shen et al., 2012). As the first step in characterizing the *in vivo* role of LZK in mammalian CNS injury response, the expression pattern of endogenous LZK was examined using immunostaining following dorsal spinal cord crush injury (see Experimental Procedures). In the uninjured spinal cord, low baseline expression of the astrocyte marker glial fibrillary astrocyte protein (GFAP) and LZK were detectable by immunofluorescence (Figure 1A). Two weeks after injury, GFAP was upregulated in astrocytes as expected (Burda and Sofroniew, 2014; Sofroniew, 2014) (Figure 1A). Concurrently, LZK immunoreactivity was markedly increased in the perilesional region and co-labeled with GFAP, especially in the gray matter (Figures 1A and S1A). These observations indicate that injury increases LZK expression in astrocytes and raise the possibility that LZK may be functionally involved in the astrocytic response to CNS injury.

Inducible LZK Deletion in Adult Astrocytes Impairs Astrogliosis and Glial Scar Formation after Spinal Cord Injury

To test the role of astrocytic LZK in astrogliosis after CNS injury, we generated tamoxifen-inducible, astrocyte-specific LZK knockout mice (GFAP-CreER^{T2};LZK$^{f/f}$ [LZK conditional knockout]) along with LZK$^{f/f}$ littermate controls (Figures 1B and 1C). We administered tamoxifen to adult mice at ages 8–10 weeks during a 5-day period and waited for another week before inducing spinal cord injury (see Experimental Procedures). Following tamoxifen treatment, GFAP expression in the spinal cords of uninjured mice was comparable between control (LZK$^{f/f}$) and astrocytic LZK knockout (GFAP-CreER^{T2};LZK$^{f/f}$) mice (Figure S1). After spinal cord injury, astrocytic LZK expression was induced in the spinal cords of LZK$^{f/f}$ control mice but not in GFAP-CreER^{T2};LZK$^{f/f}$ mice, verifying efficient deletion of LZK in astrocytes (Figures 1D and S1B).

Focal trauma to the spinal cord in mice results in a GFAP-sparse fibrotic lesion core surrounded by a GFAP-dense astroglial scar, which represents an extreme form of astrogliosis that gradually tapers off at increasing distances from the lesion core (Burda and Sofroniew, 2014). At 2 weeks after spinal cord injury, at which time the glial scar is considered mature based on previous studies in mice (Herrmann et al., 2008; Herrmann et al., 2010; Wanner et al., 2013), we analyzed the status of

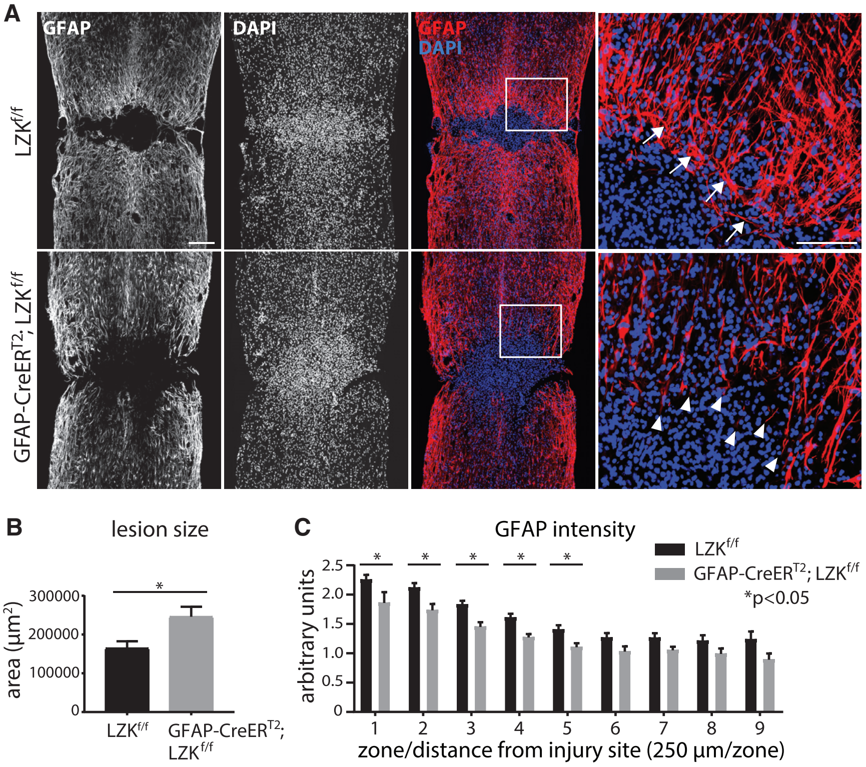

Figure 2. LZK Deletion in Adult Astrocytes Impaired Astrogliosis 14 Days after Spinal Cord Injury

(A) Representative images of GFAP and DAPI nuclear staining centered at the spinal cord injury site from tamoxifen-pretreated $LZK^{f/f}$ control and GFAP-$CreER^{T2}$;$LZK^{f/f}$ mice. Note that the $GFAP^-$ lesion core is enveloped by $GFAP^+$ astrocytes and their processes. Areas within the white boxes are shown in high magnification (rightmost panels) to illustrate astrocytic processes parallel (arrows) to the lesion border in control mice but perpendicular (arrowheads) in astrocytic LZK knockout mice. Figures are composites of smaller microscopy images. Scale bar represents 200 μm (low magnification) and 100 μm (high magnification).

(B) Quantification of lesion size in $LZK^{f/f}$ control versus GFAP-$CreER^{T2}$;$LZK^{f/f}$ mice. n = 8 mice per genotype, *$p < 0.05$ by unpaired parametric t test.

(C) GFAP immunofluorescence intensity in the injured spinal cords of $LZK^{f/f}$ control versus GFAP-$CreER^{T2}$;$LZK^{f/f}$ mice in 9 zones (each the width of the spinal cord and length of 250 μm). Zone 1 starts at the lesion border, followed by the other zones placed sequentially away from the injury site and immediately adjacent to one another, as adapted from Wanner et al., (2013). n = 8 mice per genotype, *$p < 0.05$ by two-way ANOVA followed by post hoc multiple t test between groups for each zone.

Error bar represents SEM.

astrogliosis by its prominent features as follows: maturation of the scar border as assessed by orientation of astrocytic processes together with lesion size, upregulation of cytoskeletal proteins GFAP and vimentin, and astrocyte proliferation (Sofroniew, 2014). In tamoxifen-treated $LZK^{f/f}$ control mice, cellular processes of astrocytes were predominantly oriented parallel to the fibrotic-astroglial border (Figure 2A). In mice depleted of astrocytic LZK, astrocytes at the lesion border formed a less compact scar border with astrocytic processes often perpendicular to the lesion border (Figure 2A), which is characteristic of impaired astrocyte-fibroblast segregation and scar formation. Correspondingly, lesion size was increased by ~50% in mice lacking astrocytic LZK (Figure 2B). In control mice, spinal cord injury resulted in an increase of ~2- to 3-fold in GFAP immunoreactivity immediately adjacent to the lesion core, and GFAP upregulation was diminished toward baseline at ~1.5 mm away from the injury site (Figure 2C). In comparison, within the same region, such injury-dependent GFAP upregulation was diminished by ~20% in mice lacking astrocytic LZK (Figure 2C). Likewise, injury-induced upregulation of vimentin, known to occur in reactive astrocytes (Zamanian et al., 2012), was reduced by ~20% immediately surrounding the lesion core in mice lacking astrocytic LZK (Figures 3A and 3C).

CNS injury induces astroglial proliferation within 2 weeks following trauma, with the highest number of proliferating astrocytes present closest to the lesion site following spinal cord injury (Burda and Sofroniew, 2014; Sofroniew, 2014; Wanner et al., 2013). We identified proliferating astrocytes based on immunofluorescence co-labeling of the cell proliferation marker Ki67 and the astroglial nuclear marker SOX9 (Sun et al., 2017) (Figure 3B). Seven days after spinal cord injury, the number of $Ki67^+SOX9^+$ cells immediately surrounding the lesion site was decreased by ~40% in mice lacking LZK in adult astrocytes as compared to control mice (Figures 3B and 3D). We also examined astroglial proliferation following bromodeoxyuridine (BrdU) incorporation into $GFAP^+$ cells (Wanner et al., 2013) and observed a 25% reduction in $BrdU^+GFAP^+$ cell number surrounding the injury site in mice lacking astrocytic LZK (Figure S2). Taken together, these observations indicate that following spinal cord injury, loss of LZK in astrocytes impaired astrogliosis as assessed by its hallmarks of GFAP/vimentin upregulation, astroglial scar formation, and astrocyte proliferation, thereby supporting LZK as an important regulator of reactive astrogliosis.

Inducible LZK Overexpression in Adult Astrocytes Enhances Reactive Astrogliosis after Spinal Cord Injury

To complement the LZK loss-of-function analyses above, we next conducted analyses of genetic gain of function for LZK after CNS injury. To do this, we generated mice that overexpress LZK in astrocytes upon tamoxifen treatment (GFAP-$CreER^{T2}$;LZK^{OE}) (Figures 4A and 4B). In this transgenic line, the LZK coding sequence is linked to that of the red fluorescent protein tdTomato (tdT) through the T2A peptide, thereby enabling fluorescent identification of LZK-overexpressing astrocytes. We treated 8- to 10-week-old mice with tamoxifen and then induced spinal cord injury (see Experimental Procedures). We were intrigued that mice with astrocyte-specific LZK overexpression had poor survival, with only 12% survival after 2 weeks post-injury

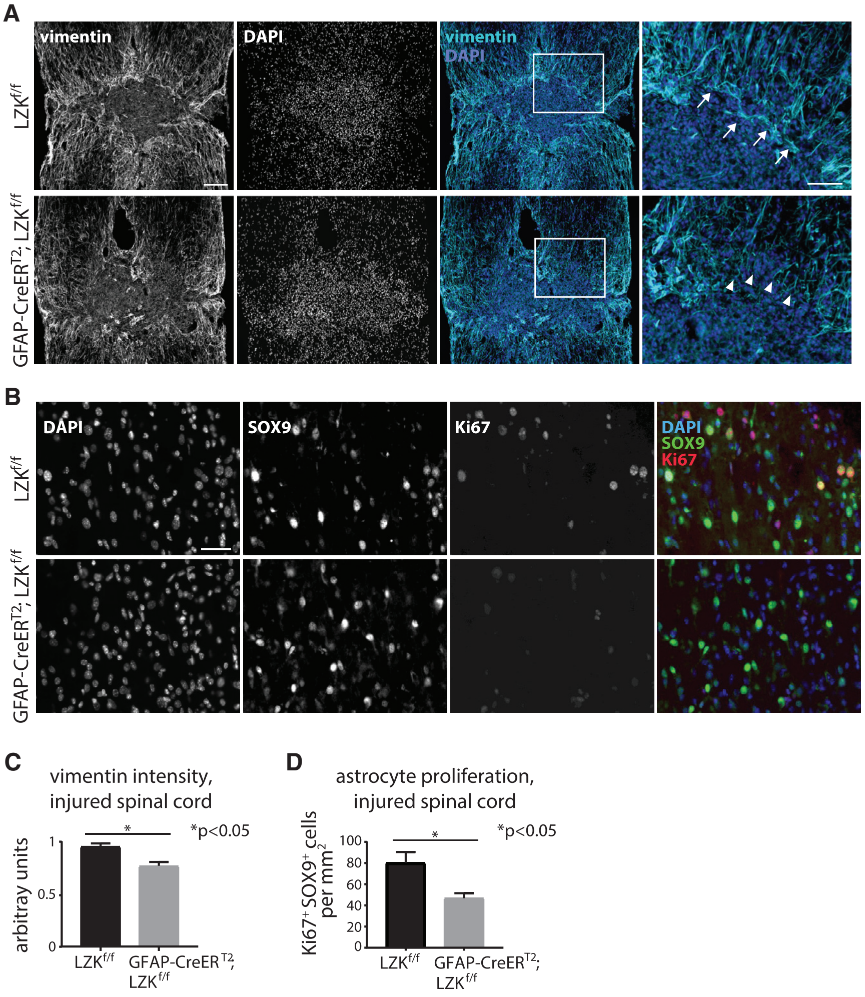

Figure 3. LZK Deletion in Adult Astrocytes Reduced Astrogliosis in the Injured Spinal Cord as Assessed by Vimentin Expression and Astrocyte Proliferation

(A) Representative images of vimentin and DAPI staining at the spinal cord injury site of tamoxifen-treated $LZK^{f/f}$ control and GFAP-$CreER^{T2}$;$LZK^{f/f}$ mice 14 days post-injury (dpi). Astrocytes strongly expressing vimentin enclose the lesion core. Areas within white boxes are shown in high magnification (rightmost panels) to illustrate astrocytic processes parallel (arrows) and perpendicular (arrowheads) to the lesion border in control mice and mice lacking astrocytic LZK, respectively. Figures are composites of smaller microscopy images.

(B) Representative images of DAPI, SOX9, and Ki67 co-immunofluorescence staining within 250 μm of the spinal cord injury site of tamoxifen-treated $LZK^{f/f}$ control and GFAP-$CreER^{T2}$;$LZK^{f/f}$ mice at 7 dpi.

(C) Quantification of vimentin immunofluorescence intensity at and within 250 μm of the lesion border in control versus astrocytic LZK knockout mice 14 dpi. n = 4 per genotype, $*p < 0.05$ by two-tailed unpaired parametric t test.

(D) Quantification of the numbers of proliferating astrocytes by $Ki67^{+}SOX9^{+}$ co-labeling within 250 μm of spinal cord injury site in control versus astrocytic LZK knockout mice 7 dpi. n = 3 per genotype, $*p < 0.05$ by two-tailed unpaired parametric t test.

Scale bars, 200 μm (A, low magnification), 100 μm (A, high magnification, rightmost panels), and 50 μm (B). See also Figure S2.

(n = 26), compared to a 95% survival rate for control mice (n = 35) that underwent the same procedures. Astrocytic LZK-overexpressing mice lost weight after tamoxifen treatment, even before spinal cord injury. The cause of death remains to be determined.

In the injured spinal cords of astrocytic LZK-overexpressing mice, a subset of $GFAP^{+}$ astrocytes were tdT^{+} with intensely upregulated LZK (Figure 4C), demonstrating induction of the LZK-tdT transgene. As expected, control mice (tamoxifen-treated LZK^{OE} mice without GFAP-$CreER^{T2}$) did not exhibit any tdT expression (Figure 4C). Consistent with previous observations (Herrmann et al., 2008; Wanner et al., 2013), astrocytes formed a compact scar 2 weeks after spinal cord injury in control mice (Figure 4D). Astrocytic LZK-overexpressing mice also exhibited a compact scar (Figure 4D). However, the average lesion size in mice with astrocytic LZK overexpression was reduced to ~60% of that of the control mice, indicating that astrocytic LZK overexpression led to a more compact injury site (Figure 4E). It is striking that at the site of injury, LZK-overexpressing astrocytes lined the lesion border, as depicted by the presence of GFAP and tdT double-positive ($GFAP^{+}tdT^{+}$) astrocytes and their processes enveloping the lesion core (Figure 4D, bottom panels). Immediately surrounding the lesion core, both LZK-overexpressing and control mice showed similar levels of GFAP expression levels (Figure 4F, zone 1). However, at 1.5 mm from the injury site, where GFAP signal intensity typically tapered toward baseline in the control group (38% of peak GFAP expression as measured at the scar border), GFAP intensity was sustained at ~70% of peak levels in mice overexpressing LZK (Figure 4F, zone 6). Even beyond 2 mm from the lesion, astrocytic LZK-overexpressing mice exhibited an almost 2-fold increase in GFAP levels compared to the control mice (Figure 4F, zone 9). Consistent with this, in LZK-overexpressing mice, astrocytes near the injury site displayed morphologies of augmented hypertrophy, compared to control mice after spinal cord injury (Figure 4C). A different surgeon was able to reproduce this key finding of enhanced astrogliosis, leading to a more compact injury site in astrocytic LZK-overexpressing mice (Figures S3A–S3C). LZK-tdT-overexpressing astrocytes densely decorated the injury site and exhibited elongated processes (Figure S3B), as previously ascribed to reactive astrocytes forming the glial scar border (Wanner et al., 2013).

In summary, mice overexpressing astrocytic LZK reproducibly exhibited enhanced astrogliosis and a more compact injury site after spinal cord injury. Corroborating our results with the astrocyte-specific LZK knockout mice described above, these data indicate that LZK promotes astrogliosis and glial scar formation after CNS injury.

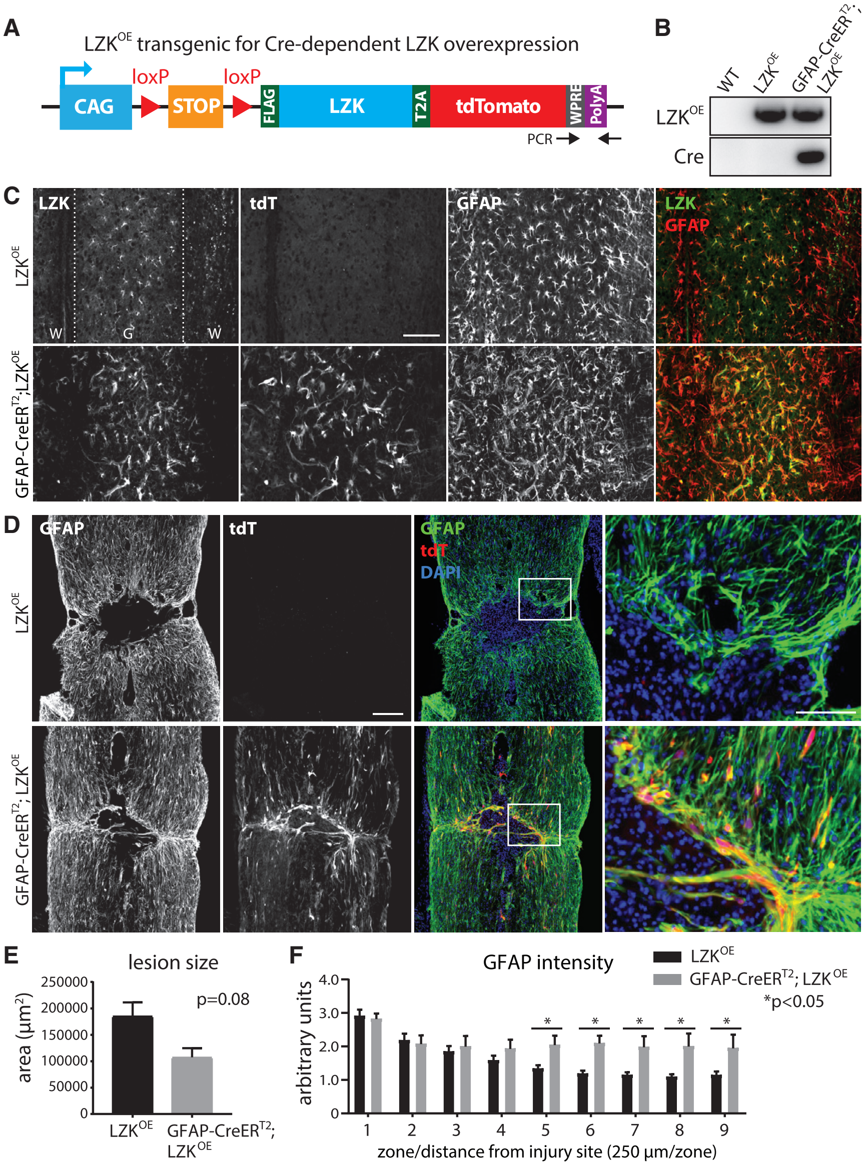

Figure 4. LZK Overexpression in Adult Astrocytes Enhanced Astrogliosis and Reduced Lesion Size 14 dpi

(A) Diagram of the LZK conditional overexpression transgene (LZK^{OE}). LZK^{OE} has two *loxP* sites flanking a STOP cassette upstream of an LZK-T2A-tdTomato (tdT) fusion gene. Cre-mediated excision of STOP would activate LZK-T2A-tdT, leading to overexpression of LZK and the associated fluorescent reporter tdT. Black arrows mark the positions of PCR primers for genotyping.

(B) Genomic PCR genotyping of WT, LZK^{OE}, and GFAP-$CreER^{T2}$;LZK^{OE} mice.

(C) Representative images taken from perilesional area (0.5–1 mm from the injury site), showing that tdT activation was associated with LZK overexpression, GFAP upregulation, and astrocyte hypertrophy in tamoxifen-pretreated GFAP-$CreER^{T2}$;LZK^{OE} mice as compared with LZK^{OE} control mice. Note LZK and GFAP co-localization in the merged panels.

(D) Representative images of GFAP immunostaining, tdT direct fluorescence, and DAPI nuclear staining centered at the injury site in the spinal cords of tamoxifen-pretreated LZK^{OE} control mice and GFAP-$CreER^{T2}$;LZK^{OE} mice. Areas within the white boxes are shown in high magnification (rightmost panels) to illustrate the lesion borders lined by $GFAP^{+}$ astrocytes. Note the presence of tdT^{+} astrocytes and the more compact lesion in the GFAP-$CreER^{T2}$;LZK^{OE} mouse. Figures are composites of smaller microscopy images. Scale bar represents 250 μm (low magnification), 100 μm (high magnification).

(E) Quantification of lesion size in LZK^{OE} control versus GFAP-$CreER^{T2}$;LZK^{OE} mice. n = 5 for LZK^{OE} mice; n = 3 for GFAP-$CreER^{T2}$;LZK^{OE} mice; p = 0.08 by unpaired parametric t test. See Figure S3 for an independent replicate experiment by a second surgeon.

(F) GFAP immunofluorescence intensity in the injured spinal cords of $LZK^{f/f}$ control versus GFAP-$CreER^{T2}$;$LZK^{f/f}$ mice in 9 zones defined similarly in Figure 2C. Same numbers of mice as in (E); *$p < 0.05$ by two-way ANOVA followed by post hoc multiple t test between groups for each zone. Error bar represents SEM.

See also Figure S3.

LZK Overexpression in Adult Astrocytes Alone Induces Widespread Astrogliosis in the Absence of CNS Injury

Given that overexpressing LZK in astrocytes enhanced astrogliosis after spinal cord injury, we next asked whether such an effect is dependent on injury. Three weeks after the end of tamoxifen treatment in adult mice, we examined GFAP expression in the CNS of controls (LZK^{OE}) and mice overexpressing astrocytic LZK (GFAP-$CreER^{T2}$;LZK^{OE}). In the spinal cords of uninjured controls, baseline GFAP expression was detectable in the spinal cords and especially in the white matter by immunofluorescence (Figures 5A and 5B). Following induction of astrocyte-specific LZK overexpression, we observed a marked increase in GFAP immunoreactivity throughout the spinal cord, with the most pronounced upregulation detected in the gray matter, where only a low level of baseline GFAP immunoreactivity was present in the control group (Figures 5A and 5B). Quantitative analyses revealed that astrocyte-specific LZK overexpression led to an ~2-fold increase in GFAP immunoreactivity in the spinal cords of uninjured mice (Figure 5C). At higher magnifications, co-localization of induced LZK and tdT along with upregulated GFAP could be discerned clearly in the spinal cords of uninjured GFAP-$CreER^{T2}$;LZK^{OE} mice (Figure 5D).

Such upregulation of GFAP immunoreactivity in uninjured mice overexpressing astrocytic LZK was not restricted to the spinal cord. In control mice, baseline levels of GFAP immunoreactivity were observed throughout the brain, with moderately higher expression in several regions, including the hippocampus and some white matter tracts (e.g., corpus callosum) (Figure 5E). In contrast, astrocytic LZK overexpression led to a dramatic increase in GFAP immunoreactivity in the cerebral cortex, hypothalamus, and many other regions throughout the brain

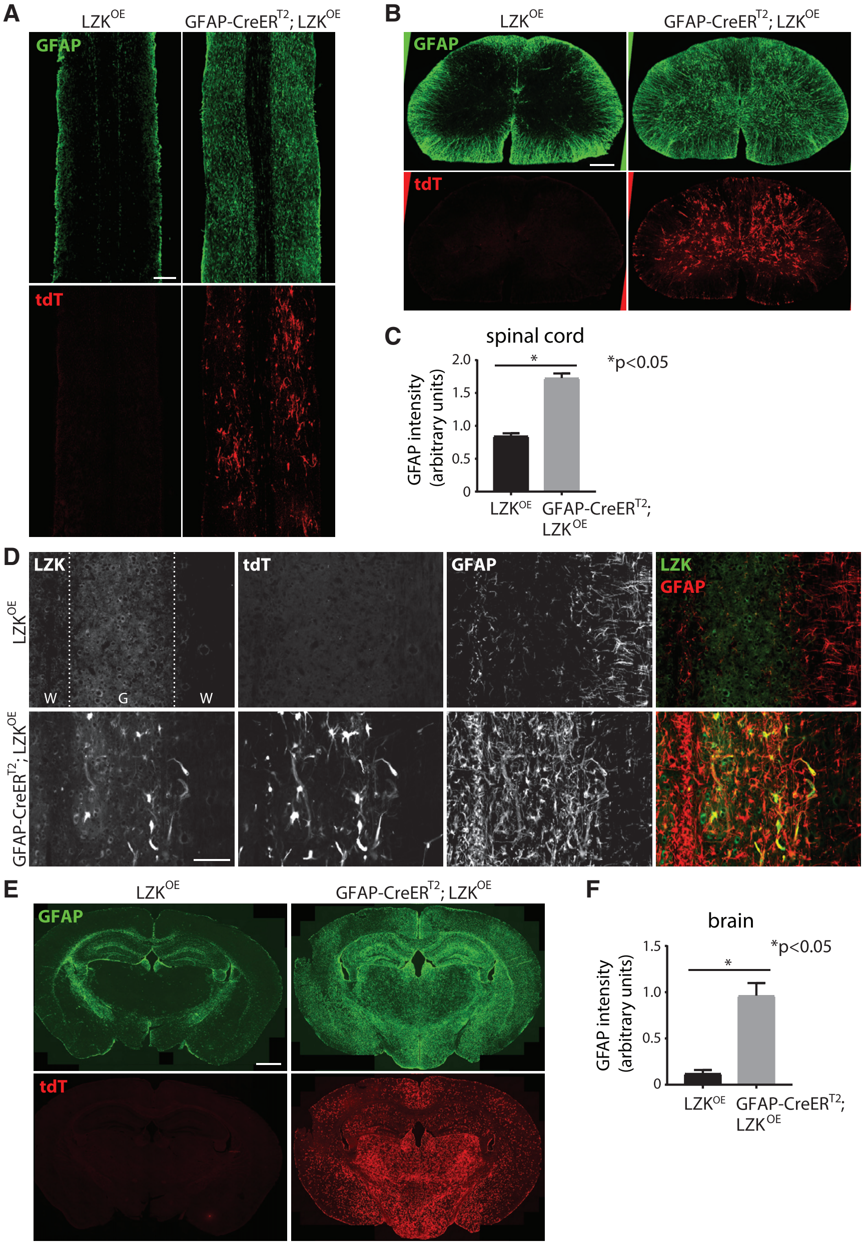

Figure 5. LZK Overexpression in Adult Astrocytes Induced Widespread Astrogliosis in the Absence of Injury as Assessed by GFAP Immunoreactivity

(A and B) Representative images of GFAP immunostaining and tdT direct fluorescence on horizontal (A) and transverse (B) spinal cord sections from tamoxifen-treated, uninjured LZK^{OE} control and GFAP-$CreER^{T2}$;LZK^{OE} mice.

(C) Quantification of GFAP immunofluorescence intensity on horizontal spinal cord sections from LZK^{OE} control versus GFAP-$CreER^{T2}$;LZK^{OE} mice after tamoxifen treatment. n = 3 mice per genotype, $^*p < 0.05$ by unpaired parametric t test.

(D) Higher magnification images of LZK, tdT, and GFAP signals on horizontal spinal cord sections from tamoxifen-treated, uninjured mice, showing co-localization of LZK/GFAP and noting similar patterns of LZK with tdT direct fluorescence. Dotted lines demarcate white (W) matter and gray (G) matter.

(E) LZK, GFAP immunostaining, and tdT direct fluorescence in the brains of tamoxifen-treated LZK^{OE} control and GFAP-$CreER^{T2}$;LZK^{OE} mice.

(F) Quantification of GFAP immunofluorescence intensity in the cerebral cortices of tamoxifen-treated LZK^{OE} versus GFAP-$CreER^{T2}$;LZK^{OE} mice. n = 3 mice per genotype, $^*p < 0.05$ by unpaired parametric t test. Error bar represents SEM.

Scale bars, 250 μm (A and B), 100 μm (D), and 1 mm (E). See also Figures S4 and S5.

(Figure 5E). As in the spinal cord, increased GFAP upregulation was most pronounced in the gray matter, likely because of the higher expression of the LZK-tdT transgene there. Quantitative analyses revealed that astrocytic LZK overexpression led to an ~10-fold increase in GFAP immunoreactivity in the cerebral cortex, where baseline levels of GFAP in control mice were low (Figure 5F). It is interesting to note that only a small subset (~13%) of these $GFAP^+$ astrocytes co-labeled with tdT, suggesting that LZK overexpression may lead to both cell-autonomous and non-cell-autonomous induction of astrocyte reactivity.

To confirm that astrocytic LZK overexpression affects general astrogliosis and not merely the expression of GFAP, we also examined vimentin expression and astroglial proliferation. In the absence of any injury, astrocytic LZK overexpression markedly increased vimentin expression and astrocyte proliferation broadly in the CNS, with the latter assessed by Ki67 and GFAP co-labeling (Figure 6). Therefore, astrocytic overexpression of LZK alone was sufficient to upregulate molecular markers of astrogliosis and to promote astroglial proliferation in the absence of any injury.

LZK Is an Upstream Activator of SOX9 and STAT3 with Mitogenic Effects

To begin identifying downstream effectors through which LZK promotes astrogliosis, we assessed the activation and expression of two key transcription factors that were previously shown to regulate the astrocytic response to spinal cord injury, SOX9 and signal transducer and activator of transcription 3 (STAT3). Genetic loss-of-function studies previously implicated SOX9 in promoting astrocyte reactivity and the upregulation of associated CSPGs (McKillop et al., 2013). SOX9 expression levels in astrocytes also are elevated after CNS insults as assessed with stroke and amyotrophic lateral sclerosis (ALS) models (Sun et al., 2017). Outside the periventricular region, the central canal, and neurogenic areas, SOX9 predominantly labels the nuclei of astrocytes (Sun et al., 2017). We performed SOX9 immunostaining and detected an ~10% increase in the number of $SOX9^+$ cells in

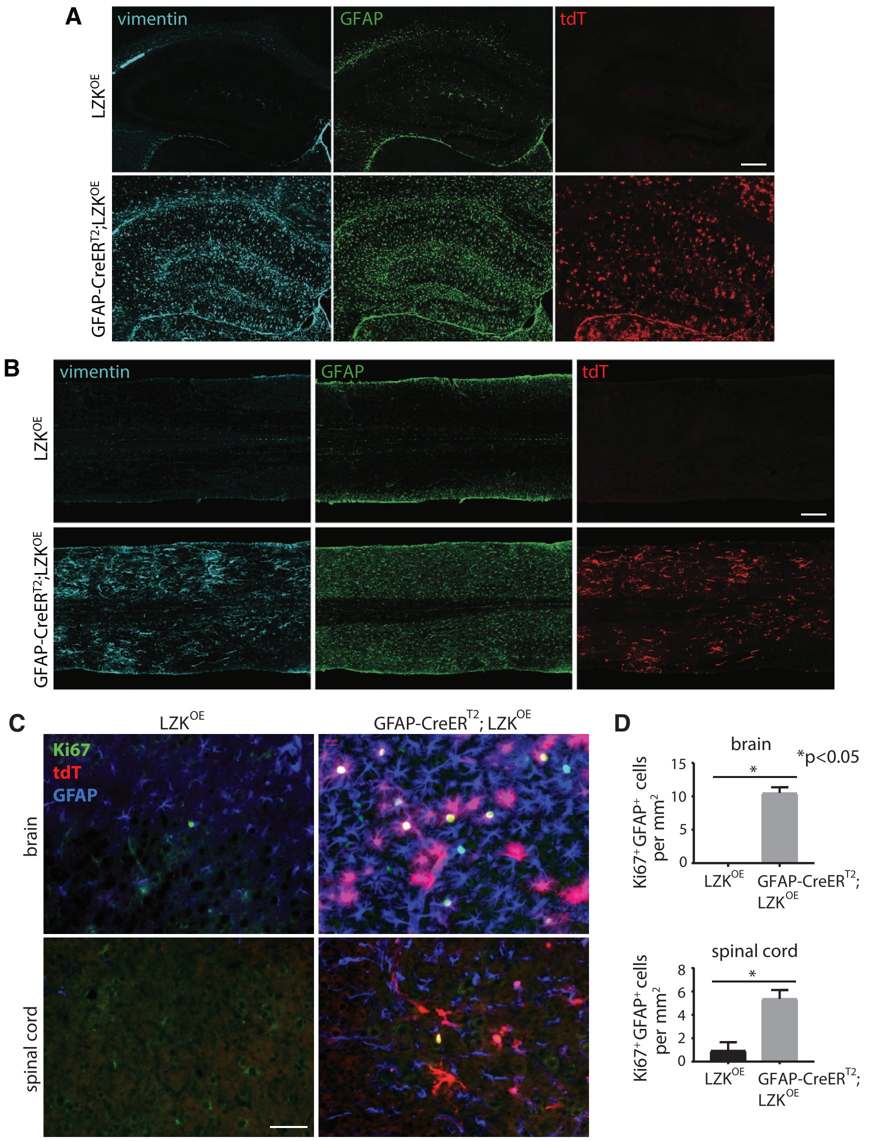

Figure 6. LZK Overexpression in Adult Astrocytes Induced Widespread Astrogliosis in the Absence of Injury as Assessed by Vimentin Immunoreactivity and Astrocyte Proliferation

(A and B) Vimentin, GFAP immunostaining, and tdT direct fluorescence on coronal sections from the hippocampal region of the brains (A) and horizontal spinal cord sections (B) of LZK^{OE} control and GFAP-$CreER^{T2}$;LZK^{OE} mice after tamoxifen treatment. Note the similarly upregulated vimentin and GFAP immunoreactivity in GFAP-$CreER^{T2}$;LZK^{OE} mice.

(C) Ki67, GFAP immunostaining, and tdT direct fluorescence in the cerebral cortices (brain) and spinal cord gray matter from LZK^{OE} control and GFAP-$CreER^{T2}$;LZK^{OE} mice.

(D) Quantification of $Ki67^{+}GFAP^{+}$ cell numbers in the cerebral cortices (brain) and spinal cord gray matter of LZK^{OE} control and GFAP-$CreER^{T2}$;LZK^{OE} mice. n = 3 per group, p value determined by unpaired parametric t test. Error bar represents SEM. Scale bars 250 μm (A and B) and 50 μm (C). (A) and (B) are composites of smaller microscopy images.

the cerebral cortices and spinal cords of uninjured mice overexpressing astrocytic LZK (Figures 7A–7D). This was accompanied by an ~2-fold increase in SOX9 immunofluorescence intensity per cell (Figures 7A–7D). Such an increase in $SOX9^{+}$ cell number was likely the result of increased astrocyte cell proliferation (Figures 6C and 6D). Thus, in the absence of any injury, overexpressing LZK in astrocytes alone was sufficient to elevate both SOX9 levels in astrocytes and the number of $SOX9^{+}$ cells.

STAT3, another astrogliosis-associated transcription factor that acts in the Janus kinase (JAK)-STAT signaling pathway, has been shown to mediate injury-dependent astrogliosis and glial scar formation (Herrmann et al., 2008; Okada et al., 2006). Activation of STAT3 can be detected by phosphorylation at tyrosine 705 (Herrmann et al., 2008), hereafter referred to as pSTAT3. We found few $pSTAT3^{+}$ cells or $pSTAT3^{+}GFAP^{+}$ astrocytes in the spinal cords of uninjured control mice (Figures 7E, S3D, and S3E). In contrast, uninjured mice with astrocyte-specific LZK overexpression had an ~10-fold increase in the number of $pSTAT3^{+}$ cells and $pSTAT3^{+}$ astrocytes (Figures 7E, S3D, and S3E), some of which co-labeled with tdT (Figure 7E). After spinal cord injury, increased numbers of $pSTAT3^{+}$ cells and $pSTAT3^{+}$ astrocytes also were observed at the injury site and perilesional region in mice overexpressing astrocytic LZK, as compared with injured control mice (Figures S3A, S3B, S3D, and S3E).

Previously, we identified JNK as the main downstream effector of the LZK signal pathway promoting axon outgrowth in cultured CNS neurons (Chen et al., 2016). Curiously, here, JNK activation was rarely detected in LZK-overexpressing astrocytes in the absence of injury *in vivo* (Figure S4). Notably, overexpressing LZK in parvalbumin (Pv)-positive neurons using the same inducible transgenic overexpression line (Pv-Cre;LZK^{OE}) did not cause animal death or alteration in the gross morphology of Pv-expressing neurons in the cerebellum (Figure S5), arguing against non-specific, toxic effects of LZK overexpression.

In summary, these data suggest that LZK promotes astrogliosis by upregulating SOX9 and activating STAT3, two known regulators of astrocyte reactivity. Furthermore, JNK does not appear to be a robust target of LZK in adult astrocytes, suggesting cell-type-specific and context-dependent LZK signaling.

DISCUSSION

Reactive astrocytes are recognized increasingly for their important contributions to injury response, disease pathogenesis, plasticity, and repair in the CNS (Pekny et al., 2016). A better

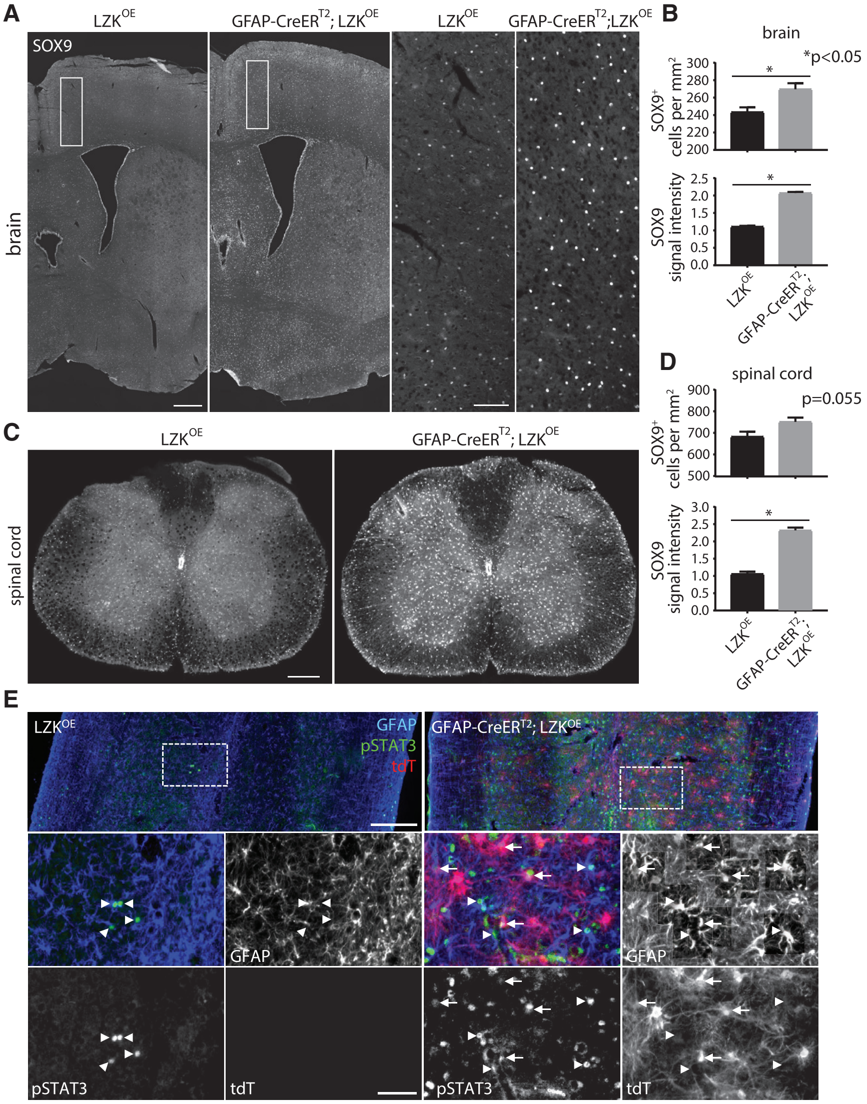

Figure 7. LZK Positively Regulates SOX9 and pSTAT3 in Adult Astrocytes *In Vivo*

(A) Immunofluorescence staining of SOX9 on coronal brain sections from LZK^{OE} control and GFAP-$CreER^{T2}$;LZK^{OE} mice. Lower-magnification images are shown in the left panels, with cerebral cortical areas within the white boxes shown in high magnification in the right panels. Note the higher levels of SOX9 immunoreactivity in GFAP-$CreER^{T2}$;LZK^{OE} mice.

(B) Quantification of $SOX9^+$ cell number and SOX9 immunofluorescence intensity in the cerebral cortices of LZK^{OE} control and GFAP-$CreER^{T2}$;LZK^{OE} mice. n = 3 per group, p value determined by unpaired parametric t test.

(C) Immunofluorescence staining of SOX9 on transverse spinal cord sections. Note the higher levels of SOX9 immunoreactivity in GFAP-$CreER^{T2}$;LZK^{OE} mice.

(D) Quantification of $SOX9^+$ cell number and SOX9 immunofluorescence intensity in the spinal cord gray matter of LZK^{OE} control and GFAP-$CreER^{T2}$;LZK^{OE} mice. n = 3 per group, p value determined by unpaired parametric t test.

(E) Immunofluorescence detection of GFAP, pSTAT3, and tdT on horizontal spinal cord sections of LZK^{OE} control (left panels) and GFAP-$CreER^{T2}$;LZK^{OE} mice (right panels). Arrows represent tdT/pSTAT3 co-labeled cells; arrowheads represent GFAP/pSTAT3 co-labeled cells but without tdT co-labeling. Quantification of these data (shown in Figures S3D and S3E) indicates that LZK overexpression in adult astrocytes increased $pSTAT3^+$ cells/astrocytes in the uninjured spinal cord.

Scale bars, 500 μm (A, low magnification), 100 μm (A, high magnification), 250 μm (C), 200 μm (E, low magnification), 50 μm (E, high magnification). (A), (C), and (E) are composites of smaller microscopy images.

understanding of the molecular underpinning of reactive astrogliosis provides valuable tools to manipulate this process, unravel its complex functions, and affect the outcome of a multitude of neurological conditions. Our study revealed LZK to be a positive, cell-intrinsic regulator of astrogliosis, as evidenced by its ability to promote the expression of reactive astrocyte markers, astrocyte cell proliferation, and maturation of the glial scar. Notably, overexpressing LZK in astrocytes alone was sufficient to drive widespread astrogliosis throughout the otherwise unperturbed CNS. These data support LZK as a potential target of astroglial manipulation for improving the outcome in a spectrum of CNS injuries and diseases.

Genetic loss and gain of LZK function in astrocytes decreased and increased astrogliosis, respectively, as assessed by multiple established features defining astrogliosis, demonstrating LZK as a cell-intrinsic regulator of astrocyte reactivity. However, the effect of LZK overexpression appeared to "spill over" to other astrocytes: although only a relatively small subset of astrocytes (~13%) in GFAP-$CreER^{T2}$;LZK^{OE} mice overexpressed LZK, as assessed by expression of its reporter tdT, GFAP was systemically upregulated throughout the CNS, even in the absence of an injury or other insult. Thus, LZK may promote astrogliosis both cell autonomously and non-cell autonomously. It remains to be excluded, however, that LZK-overexpressing astrocytes may simply give rise to progeny that do not express the LZK-tdT transgene. Following spinal cord injury, LZK-overexpressing astrocytes were found to line the lesion border with an elongated morphology, which is consistent with the possibility that LZK regulates the astrocyte response to a gradient of instructional cues emanating from the lesion core (Burda and Sofroniew, 2014). The effects of astrocytic LZK overexpression on other injury responses such as blood-brain barrier permeability and the spread of inflammation remain to be investigated.

STAT3, SOCS3 (repressor of STAT3), and nuclear factor κB (NF-κB) are among the key molecules previously shown to regulate astrogliosis and lesion size after spinal cord injury (Brambilla

et al., 2005; Herrmann et al., 2008; Okada et al., 2006). SOCS3 deletion enhances glial scar formation and wound healing after spinal cord injury; however, it is not known whether it is sufficient to drive astrogliosis in the otherwise unperturbed CNS (Okada et al., 2006). The ability to induce astrogliosis with LZK overexpression alone in the absence of any injury or other insult would provide a new avenue to study the pathophysiological roles of astrogliosis in CNS injury and disease (Pekny et al., 2016). Furthermore, LZK overexpression in astrocytes led to a widespread upregulation of pSTAT3, again suggesting that LZK activates the JAK-STAT pathway in both a cell-autonomous and non-cell-autonomous manner. Likewise, astrocytic LZK overexpression upregulated the expression of SOX9, another molecule that is implicated in astrocyte reactivity (McKillop et al., 2013). Future studies are required to dissect the functional interaction between LZK and other signaling pathways in regulating astrocytic reactivity. In contrast to the activation of JNK (assessed by phospho-JNK [pJNK] levels) by LZK in CNS neurons (Chen et al., 2016), pJNK was rarely detected in LZK-overexpressing astrocytes. Instead, robust activation of STAT3 and upregulation of SOX9 were observed in astrocytes overexpressing LZK *in vivo*. This suggests cell-type-specific LZK signaling cascades in response to neural injury. Our work identifies LZK as a positive regulator of SOX9 and STAT3 pathways in the context of astroglial reactivity.

This work also raised an important question regarding the functional consequences of LZK-regulated astrogliosis on neural repair, especially considering that the role of reactive astrocytes and the glial scar in axonal regeneration has been investigated intensively (Anderson et al., 2016; Hara et al., 2017; Silver, 2016). Although LZK-regulated astrogliosis appears to promote wound healing, toxicity from systemic LZK overexpression in astrocytes has hindered our assessment of the impact on post-injury axon dynamics or behavioral recovery, which will likely require a method to locally overexpress LZK in the spinal cord. Given their vital roles in a variety of brain regions and the heterogeneity of both healthy and reactive astrocytes (Ben Haim and Rowitch, 2017; Khakh and Sofroniew, 2015; Liddelow and Barres, 2017), it is conceivable that LZK overexpression elicits a multitude of astroglial responses with both beneficial and detrimental consequences. However, LZK overexpression in Pv-Cre;LZK^{OE} mice did not lead to animal death or any overt phenotype in Pv-expressing neurons, arguing against non-physiological effects of LZK overexpression per se. The opposite phenotypes in astrogliosis displayed by mice lacking or overexpressing astrocytic LZK further support the role of LZK as a critical regulator of the astroglial response to CNS injury.

In our previous work, we identified LZK as a neuron-intrinsic promoter of axon growth and regeneration in cultured CNS neurons (Chen et al., 2016). A similar effect of LZK on promoting axon growth also has been reported with primary retinal ganglion cells (Welsbie et al., 2017). The function of LZK in astrocytes reported here illustrates its role as an injury sensor capable of orchestrating a multicellular response to CNS injury. It would be of significant interest to explore whether LZK-dependent regulation of astrogliosis can be extended to other forms of CNS insult, including diffuse damage, neuroinflammation, and neurodegenerative diseases. Along these lines, whole-body inducible deletion of dual leucine zipper-bearing linase (DLK, or MAP3K12), a homolog of LZK, was recently reported to attenuate astrogliosis in a mouse model of ALS; however, the authors suggested that this effect was the result of an indirect effect of neuronal DLK (Le Pichon et al., 2017), because the evolutionarily conserved role of DLK in neuronal response to injury is well documented (Hammarlund et al., 2009; Miller et al., 2009; Shin et al., 2012; Watkins et al., 2013; Welsbie et al., 2013; Yan et al., 2009). Regardless, a multicellular role for the same signaling molecule in mediating CNS response to insult underscores the importance of thoroughly understanding its cell-type-specific functions before effective translational strategies can be envisioned. The identification of LZK as a cell-intrinsic signaling molecule regulating astroglial reactivity will allow for the testing of important hypotheses regarding the complex roles of astrocyte reactivity in CNS injury and disease.

EXPERIMENTAL PROCEDURES

Genetically Modified Mice

All mouse husbandry and experimental procedures were approved by the Institutional Animal Care and Use Committee at the University of California San Diego and the University of Texas Southwestern Medical Center. Both male and female mice, ages 8–10 weeks, were used. LZK-targeted mutant mice were generated through the UC San Diego Transgenic Mouse and Gene Targeting Core using a mouse embryonic stem cell line obtained from the Knockout Mouse Project (KOMP) Repository as described previously (Chen et al., 2016). LZK$^{f/f}$ mice were generated by crossing mice carrying the LZK-targeted allele to mice expressing germline flippase (FLP) recombinase (Rodríguez et al., 2000), followed by removal of FLP recombinase by breeding to wild-type C57BL/6 mice. LZK^{f} has two *loxP* sites flanking exon 2 where Cre-mediated excision is expected to result in a frameshift and thus a null allele. These mice were crossed with the GFAP-CreER^{T2} line (Hirrlinger et al., 2006) to generate GFAP-CreER^{T2};LZK$^{f/f}$ mice in C57BL/6 background (N > 10) for this study. Transgenic LZK conditional overexpressing mice (LZK^{OE}) in FVB background were custom made by Applied StemCell. After breeding to C57BL/6 for two generations, LZK^{OE} mice were crossed with GFAP-CreER^{T2} mice (Hirrlinger et al., 2006) in C57BL/6 background to generate the GFAP-CreER^{T2};LZK^{OE} line (see illustration of LZK^{f} and LZK^{OE} alleles in Figures 1 and 4 for more details). LZK^{OE} mice were crossed with Pv-Cre mice (Hippenmeyer et al., 2005) to generate the Pv-Cre;LZK^{OE} line. Pv-Cre knockin allele has the endogenous Pv promoter/enhancer elements directing the expression of Cre recombinase, which will allow LZK overexpression in Pv^{+} cells.

General *In Vivo* Experimental Design

All of the surgical experiments, tissue processing, data acquisition, and quantification were performed by a laboratory member blinded to genotype. For experiments testing the effects of astrocyte-specific LZK deletion, 8- to 10-week-old GFAP-CreER^{T2};LZK$^{f/f}$ mice and their LZK$^{f/f}$ littermate controls each were given 75 mg/kg/day tamoxifen by oral gavage for a total of 5 gavages (days 1, 2, 3, 4, and 5). For experiments testing the effects of astrocyte-specific LZK overexpression, 8- to 10-week-old GFAP-CreER^{T2};LZK^{OE} mice and their LZK^{OE} littermate controls each were given 75 mg/kg tamoxifen by oral gavage every other day (days 1, 3, and 5), for a total of 3 gavages (the dosage was reduced as compared with LZK conditional knockout mice to alleviate animal loss). GFAP-CreER^{T2};LZK^{OE} mice lose weight following tamoxifen-induced LZK overexpression, leading to the death of some animals. In all of the mice, 1 week after the last tamoxifen treatment, a surgeon blinded to genotype performed spinal cord surgery. Two weeks after surgery, brains and spinal cords were collected and subsequently processed for qualitative and quantitative histological evaluation. Along the same timeline, uninjured mice were perfused and analyzed 3 weeks after the last tamoxifen treatment.

Surgical Procedures

All of the animals underwent general anesthesia by intraperitoneal injection of ketamine and xylazine. Using a surgical microscope (Zeiss OPMI 1FC), laminectomy of one vertebra at thoracic level T8-T9 was performed. We used a dorsal hemi-crush model as previously described by Chen et al., (2017). A pair of no. 5 Dumont forceps (Fine Science Tools) was then used to compress the dorsal spinal cord at a depth of 0.7 mm across the entire width of the cord for 5 s. This dorsal hemi-crush model allowed for better survival following surgery as compared with full spinal cord complete crush and thus was used throughout this study, especially given that mice overexpressing astrocyte-specific LZK exhibited reduced survival even without injury.

Statistical Analyses

Unpaired parametric t test or two-way ANOVA followed by post hoc t test using GraphPad Prism software calculated the statistical significance in differences between two groups. N represents the number of animals per genotype or treatment. For exact values of n, see the figure legends and the Supplemental Experimental Procedures.

SUPPLEMENTAL INFORMATION

Supplemental Information includes Supplemental Experimental Procedures and five figures and can be found with this article online at https://doi.org/10.1016/j.celrep.2018.02.102.

ACKNOWLEDGMENTS

This work was supported by an NIH National Research Service Award Individual Postdoctoral Fellowship (F32NS083186) to M.C., a grant from the Haggerty Foundation to M.P.G., funds from the Howard Hughes Medical Institute and the Craig H. Neilsen Foundation (award 316915) to Y.J., and grants from the NIH/National Institute of Neurological Disorders and Stroke (NS093055, NS054734, and NS047101), the Craig H. Neilsen Foundation (award 384971) and Wings for Life Spinal Cord Research Foundation (award WFL-US-27/17) to B.Z.. We thank the Whole Brain Microscopy Facility (WBMF) at the University of Texas Southwestern for assistance with microscope use. The WBMF is supported by the Texas Institute for Brain Injury and Repair.

AUTHOR CONTRIBUTIONS

M.C., M.P.G., Y.J., and B.Z. organized the study; M.C., Y.J., and B.Z. designed the experiments, analyzed the data, and wrote the manuscript; M.C. performed the majority of the experiments; C.G.G. performed surgical experiments to replicate the spinal cord injury results and to assess pSTAT3 levels; J.M.M. coordinated and directly contributed to the replication and pSTAT3 experiments; A.N. coordinated experiments, performed quantification, and tested additional outcome measures to address reviewers' comments; Y.L. led the experiments on cerebellum; M.T.N., V.S.K., X.K., C.L.S., K.I.C., and L.E. assisted in various aspects of the experiments; all authors contributed to data analyses, discussion, and the final manuscript.

DECLARATION OF INTERESTS

The authors declare no competing interests.

Received: September 20, 2017
Revised: November 8, 2017
Accepted: February 26, 2018
Published: March 27, 2018

REFERENCES

Anderson, M.A., Burda, J.E., Ren, Y., Ao, Y., O'Shea, T.M., Kawaguchi, R., Coppola, G., Khakh, B.S., Deming, T.J., and Sofroniew, M.V. (2016). Astrocyte scar formation aids central nervous system axon regeneration. Nature *532*, 195–200.

Ben Haim, L., and Rowitch, D.H. (2017). Functional diversity of astrocytes in neural circuit regulation. Nat. Rev. Neurosci. *18*, 31–41.

Bradbury, E.J., Moon, L.D., Popat, R.J., King, V.R., Bennett, G.S., Patel, P.N., Fawcett, J.W., and McMahon, S.B. (2002). Chondroitinase ABC promotes functional recovery after spinal cord injury. Nature *416*, 636–640.

Brambilla, R., Bracchi-Ricard, V., Hu, W.H., Frydel, B., Bramwell, A., Karmally, S., Green, E.J., and Bethea, J.R. (2005). Inhibition of astroglial nuclear factor kappaB reduces inflammation and improves functional recovery after spinal cord injury. J. Exp. Med. *202*, 145–156.

Burda, J.E., and Sofroniew, M.V. (2014). Reactive gliosis and the multicellular response to CNS damage and disease. Neuron *81*, 229–248.

Bush, T.G., Puvanachandra, N., Horner, C.H., Polito, A., Ostenfeld, T., Svendsen, C.N., Mucke, L., Johnson, M.H., and Sofroniew, M.V. (1999). Leukocyte infiltration, neuronal degeneration, and neurite outgrowth after ablation of scar-forming, reactive astrocytes in adult transgenic mice. Neuron *23*, 297–308.

Chen, M., Geoffroy, C.G., Wong, H.N., Tress, O., Nguyen, M.T., Holzman, L.B., Jin, Y., and Zheng, B. (2016). Leucine zipper-bearing kinase promotes axon growth in mammalian central nervous system neurons. Sci. Rep. *6*, 31482.

Chen, W., Lu, N., Ding, Y., Wang, Y., Chan, L.T., Wang, X., Gao, X., Jiang, S., and Liu, K. (2017). Rapamycin-resistant mTOR activity is required for sensory axon regeneration induced by a conditioning lesion. eNeuro *3*, ENEURO.0358-16.2016.

Faulkner, J.R., Herrmann, J.E., Woo, M.J., Tansey, K.E., Doan, N.B., and Sofroniew, M.V. (2004). Reactive astrocytes protect tissue and preserve function after spinal cord injury. J. Neurosci. *24*, 2143–2155.

Gallo, V., and Deneen, B. (2014). Glial development: the crossroads of regeneration and repair in the CNS. Neuron *83*, 283–308.

Hammarlund, M., Nix, P., Hauth, L., Jorgensen, E.M., and Bastiani, M. (2009). Axon regeneration requires a conserved MAP kinase pathway. Science *323*, 802–806.

Hara, M., Kobayakawa, K., Ohkawa, Y., Kumamaru, H., Yokota, K., Saito, T., Kijima, K., Yoshizaki, S., Harimaya, K., Nakashima, Y., and Okada, S. (2017). Interaction of reactive astrocytes with type I collagen induces astrocytic scar formation through the integrin-N-cadherin pathway after spinal cord injury. Nat. Med. *23*, 818–828.

Herrmann, J.E., Imura, T., Song, B., Qi, J., Ao, Y., Nguyen, T.K., Korsak, R.A., Takeda, K., Akira, S., and Sofroniew, M.V. (2008). STAT3 is a critical regulator of astrogliosis and scar formation after spinal cord injury. J. Neurosci. *28*, 7231–7243.

Herrmann, J.E., Shah, R.R., Chan, A.F., and Zheng, B. (2010). EphA4 deficient mice maintain astroglial-fibrotic scar formation after spinal cord injury. Exp. Neurol. *223*, 582–598.

Hippenmeyer, S., Vrieseling, E., Sigrist, M., Portmann, T., Laengle, C., Ladle, D.R., and Arber, S. (2005). A developmental switch in the response of DRG neurons to ETS transcription factor signaling. PLoS Biol. *3*, e159.

Hirrlinger, P.G., Scheller, A., Braun, C., Hirrlinger, J., and Kirchhoff, F. (2006). Temporal control of gene recombination in astrocytes by transgenic expression of the tamoxifen-inducible DNA recombinase variant CreERT2. Glia *54*, 11–20.

Khakh, B.S., and Sofroniew, M.V. (2015). Diversity of astrocyte functions and phenotypes in neural circuits. Nat. Neurosci. *18*, 942–952.

Le Pichon, C.E., Meilandt, W.J., Dominguez, S., Solanoy, H., Lin, H., Ngu, H., Gogineni, A., Sengupta Ghosh, A., Jiang, Z., Lee, S.H., et al. (2017). Loss of dual leucine zipper kinase signaling is protective in animal models of neurodegenerative disease. Sci. Transl. Med. *9*, eaag0394.

Liddelow, S.A., and Barres, B.A. (2017). Reactive astrocytes: production, function, and therapeutic potential. Immunity *46*, 957–967.

Liddelow, S.A., Guttenplan, K.A., Clarke, L.E., Bennett, F.C., Bohlen, C.J., Schirmer, L., Bennett, M.L., Münch, A.E., Chung, W.S., Peterson, T.C., et al. (2017). Neurotoxic reactive astrocytes are induced by activated microglia. Nature *541*, 481–487.

McKillop, W.M., Dragan, M., Schedl, A., and Brown, A. (2013). Conditional Sox9 ablation reduces chondroitin sulfate proteoglycan levels and improves motor function following spinal cord injury. Glia *61*, 164–177.

Miller, B.R., Press, C., Daniels, R.W., Sasaki, Y., Milbrandt, J., and DiAntonio, A. (2009). A dual leucine kinase-dependent axon self-destruction program promotes Wallerian degeneration. Nat. Neurosci. *12*, 387–389.

Okada, S., Nakamura, M., Katoh, H., Miyao, T., Shimazaki, T., Ishii, K., Yamane, J., Yoshimura, A., Iwamoto, Y., Toyama, Y., and Okano, H. (2006). Conditional ablation of Stat3 or Socs3 discloses a dual role for reactive astrocytes after spinal cord injury. Nat. Med. *12*, 829–834.

Pekny, M., Pekna, M., Messing, A., Steinhäuser, C., Lee, J.M., Parpura, V., Hol, E.M., Sofroniew, M.V., and Verkhratsky, A. (2016). Astrocytes: a central element in neurological diseases. Acta Neuropathol. *131*, 323–345.

Rodríguez, C.I., Buchholz, F., Galloway, J., Sequerra, R., Kasper, J., Ayala, R., Stewart, A.F., and Dymecki, S.M. (2000). High-efficiency deleter mice show that FLPe is an alternative to Cre-loxP. Nat. Genet. *25*, 139–140.

Sabelström, H., Stenudd, M., Réu, P., Dias, D.O., Elfineh, M., Zdunek, S., Damberg, P., Göritz, C., and Frisén, J. (2013). Resident neural stem cells restrict tissue damage and neuronal loss after spinal cord injury in mice. Science *342*, 637–640.

Shen, Y., Yue, F., McCleary, D.F., Ye, Z., Edsall, L., Kuan, S., Wagner, U., Dixon, J., Lee, L., Lobanenkov, V.V., and Ren, B. (2012). A map of the cis-regulatory sequences in the mouse genome. Nature *488*, 116–120.

Shin, J.E., Cho, Y., Beirowski, B., Milbrandt, J., Cavalli, V., and DiAntonio, A. (2012). Dual leucine zipper kinase is required for retrograde injury signaling and axonal regeneration. Neuron *74*, 1015–1022.

Silver, J. (2016). The glial scar is more than just astrocytes. Exp. Neurol. *286*, 147–149.

Silver, J., and Miller, J.H. (2004). Regeneration beyond the glial scar. Nat. Rev. Neurosci. *5*, 146–156.

Sofroniew, M.V. (2014). Astrogliosis. Cold Spring Harb. Perspect. Biol. *7*, a020420.

Sun, W., Cornwell, A., Li, J., Peng, S., Osorio, M.J., Aalling, N., Wang, S., Benraiss, A., Lou, N., Goldman, S.A., and Nedergaard, M. (2017). SOX9 is an astrocyte-specific nuclear marker in the adult brain outside the neurogenic regions. J. Neurosci. *37*, 4493–4507.

Wanner, I.B., Anderson, M.A., Song, B., Levine, J., Fernandez, A., Gray-Thompson, Z., Ao, Y., and Sofroniew, M.V. (2013). Glial scar borders are formed by newly proliferated, elongated astrocytes that interact to corral inflammatory and fibrotic cells via STAT3-dependent mechanisms after spinal cord injury. J. Neurosci. *33*, 12870–12886.

Watkins, T.A., Wang, B., Huntwork-Rodriguez, S., Yang, J., Jiang, Z., Eastham-Anderson, J., Modrusan, Z., Kaminker, J.S., Tessier-Lavigne, M., and Lewcock, J.W. (2013). DLK initiates a transcriptional program that couples apoptotic and regenerative responses to axonal injury. Proc. Natl. Acad. Sci. USA *110*, 4039–4044.

Welsbie, D.S., Yang, Z., Ge, Y., Mitchell, K.L., Zhou, X., Martin, S.E., Berlinicke, C.A., Hackler, L., Jr., Fuller, J., Fu, J., et al. (2013). Functional genomic screening identifies dual leucine zipper kinase as a key mediator of retinal ganglion cell death. Proc. Natl. Acad. Sci. USA *110*, 4045–4050.

Welsbie, D.S., Mitchell, K.L., Jaskula-Ranga, V., Sluch, V.M., Yang, Z., Kim, J., Buehler, E., Patel, A., Martin, S.E., Zhang, P.W., et al. (2017). Enhanced functional genomic screening identifies novel mediators of dual leucine zipper kinase-dependent injury signaling in neurons. Neuron *94*, 1142–1154 e1146.

Yan, D., Wu, Z., Chisholm, A.D., and Jin, Y. (2009). The DLK-1 kinase promotes mRNA stability and local translation in C. elegans synapses and axon regeneration. Cell *138*, 1005–1018.

Zamanian, J.L., Xu, L., Foo, L.C., Nouri, N., Zhou, L., Giffard, R.G., and Barres, B.A. (2012). Genomic analysis of reactive astrogliosis. J. Neurosci. *32*, 6391–6410.

Cell Reports, Volume *22*

Supplemental Information

Leucine Zipper-Bearing Kinase

Is a Critical Regulator of Astrocyte Reactivity

in the Adult Mammalian CNS

Meifan Chen, Cédric G. Geoffroy, Jessica M. Meves, Aarti Narang, Yunbo Li, Mallorie T. Nguyen, Vung S. Khai, Xiangmei Kong, Christopher L. Steinke, Krislyn I. Carolino, Lucie Elzière, Mark P. Goldberg, Yishi Jin, and Binhai Zheng

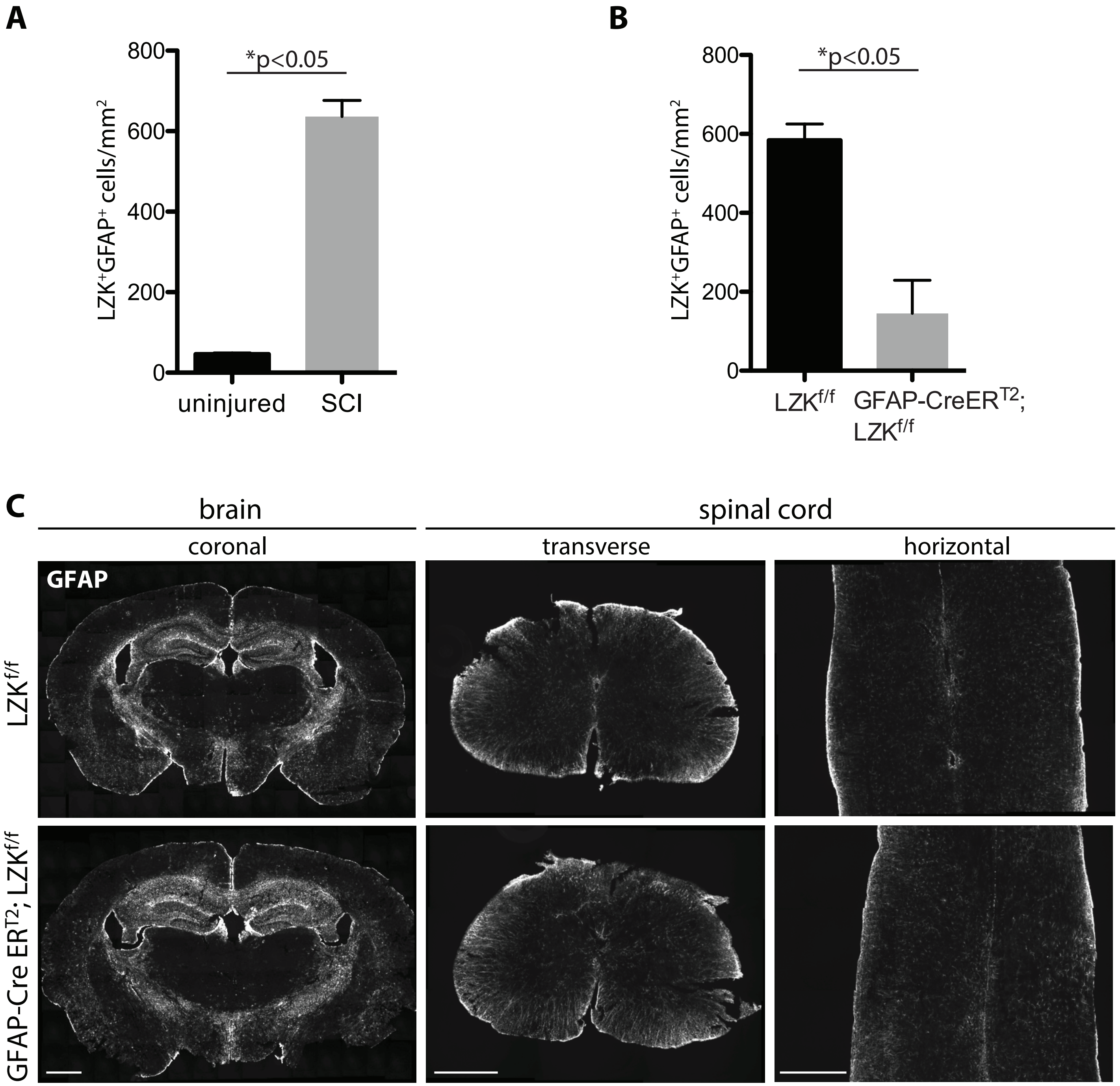

Figure S1. LZK deletion in adult astrocytes diminished injury-induced LZK upregulation and had no effect on GFAP expression in the uninjured CNS. Related to Figure 1. (A) Quantification of LZK^+GFAP^+ cell number in the uninjured spinal cords as compared to injured spinal cords of wildtype mice at 14 days post injury (dpi), perilesional area 0.5-1mm from the injury site. N=3 per condition, *$p<0.05$ by unpaired parametric t-test. Error bar, SEM. (B) Quantification of LZK^+GFAP^+ cell number in the injured spinal cords of control $LZK^{f/f}$ mice as compared to that of GFAP-$CreER^{T2}$;$LZK^{f/f}$ mice at 14 dpi, perilesional area 0.5-1mm from the injury site. N=3 per genotype, *$p<0.05$ by unpaired parametric t-test. Error bar, SEM. (C) Immunofluorescence staining of GFAP on coronal sections of the brain, and transverse and horizontal sections of the spinal cord from tamoxifen-treated $LZK^{f/f}$ control and GFAP-$CreER^{T2}$;$LZK^{f/f}$ mice. Scale bar = 1 mm (brain), 500 µm (spinal cord). Figures are composites of smaller microscopy images.

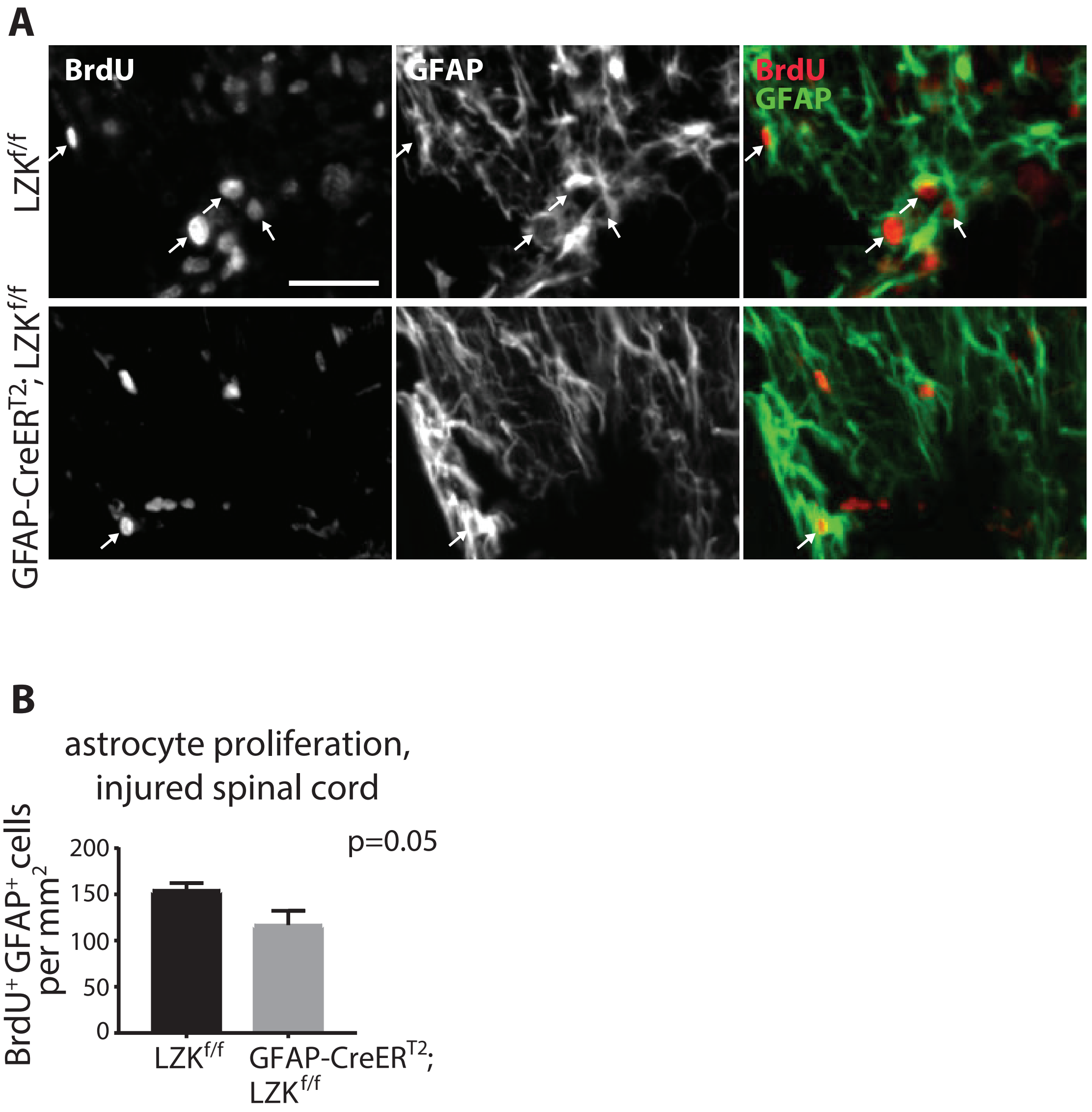

Figure S2. Reduced astrocyte proliferation in injured mice depleted of astrocytic LZK. Related to Figure 3. (A) Representative images of BrdU and GFAP co-immunofluorescence staining within 250 µm of spinal cord injury site of tamoxifen-treated $LZK^{f/f}$ control and GFAP-$CreER^{T2}$;$LZK^{f/f}$ mice sacrificed on 14 dpi. Scale bar = 50 µm. The number of BrdU+ nuclei tightly encased by or overlapping with GFAP+ astrocytic processes (arrows) is quantified in (B). Control, N=5; astrocytic LZK knockout mice, N=3. *p*=0.05 by two-tailed unpaired parametric t-test. Error bar, SEM.

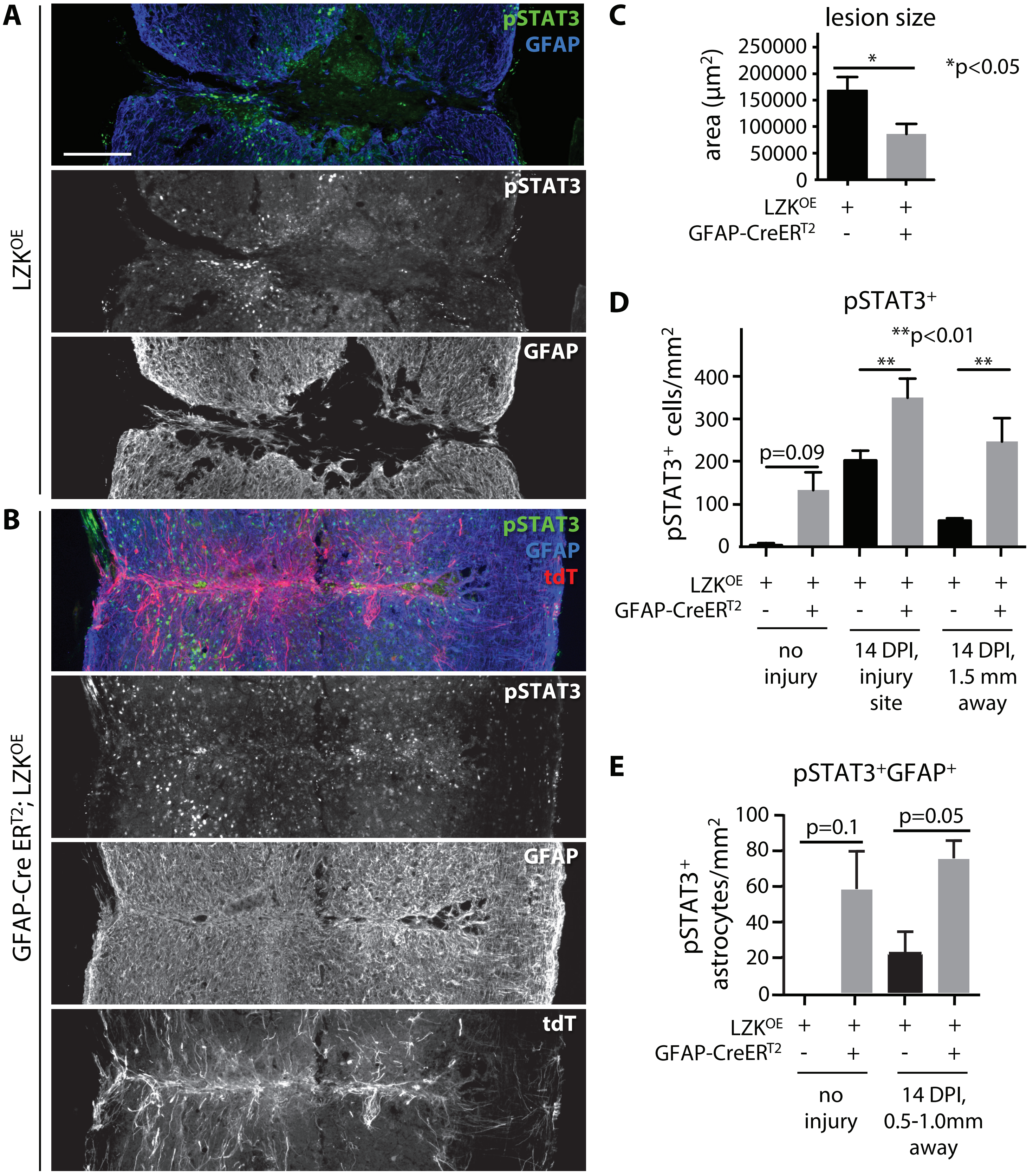

A
pSTAT3
GFAP
pSTAT3
GFAP
LZK^OE
B
pSTAT3
GFAP
tdT
pSTAT3
GFAP
tdT
GFAP-Cre ER^T2; LZK^OE
C
lesion size
area (µm²)
250000
200000
150000
100000
50000
0
*
*p<0.05
LZK^OE + +
GFAP-CreER^T2 - +
D
pSTAT3⁺
**p<0.01
pSTAT3⁺ cells/mm²
400
300
200
100
0
p=0.09
**
**
LZK^OE + + + + + +
GFAP-CreER^T2 - + - + - +
no injury
14 DPI, injury site
14 DPI, 1.5 mm away
E
pSTAT3⁺GFAP⁺
pSTAT3⁺ astrocytes/mm²
100
80
60
40
20
0
p=0.1
p=0.05
LZK^OE + + + +
GFAP-CreER^T2 - + - +
no injury
14 DPI, 0.5-1.0mm away

Figure S3

Figure S3. Confirmation of reduced injury size in mice overexpressing LZK in astrocytes in an independent surgical experiment and assessment of STAT3 activation in astrocytes in the injured spinal cord. Related to Figure 4. (A,B) Immunofluorescence detection of pSTAT3, GFAP and tdT on horizontal spinal cord sections of LZK^{OE} control (A) and GFAP-$CreER^{T2}$; LZK^{OE} mice (B) 14 days after spinal cord injury. This injury experiment was done by a different surgeon as that shown in Fig. 3. Note the elongated morphologies of tdT^+ cells and processes lining up at the injury site in GFAP-$CreER^{T2}$; LZK^{OE} mice. Scale bar = 200 µm. (C) Quantification of the lesion area 14 days post injury. (D) Quantification of the number of $pSTAT3^+$ cells in uninjured mice and 14 days post injury (at the injury site and 1.5 mm away from the lesion site). Note that LZK overexpression in astrocytes increased $pSTAT3^+$ cells in the spinal cord of both uninjured and injured mice as compared with non-overexpression controls. (E) Quantification of the number of $pSTAT3^+GFAP^+$ cells in uninjured mice and 14 days post injury (at the injury site and 1.5 mm away from the lesion site). Note that LZK overexpression in astrocytes increased the number of astrocytes with activated STAT3 (pSTAT3) in the spinal cord of both uninjured and injured mice as compared with non-overexpression controls. N = 2 per genotype (no injury); 7 (LZK^{OE} mice, 14 days post injury), 5 (GFAP-$CreER^{T2}$;LZK^{OE}, 14 days post injury), *p<0.05, **p<0.01 by unpaired parametric t-test. Error bar, SEM. Figures are composites of smaller microscopy images.

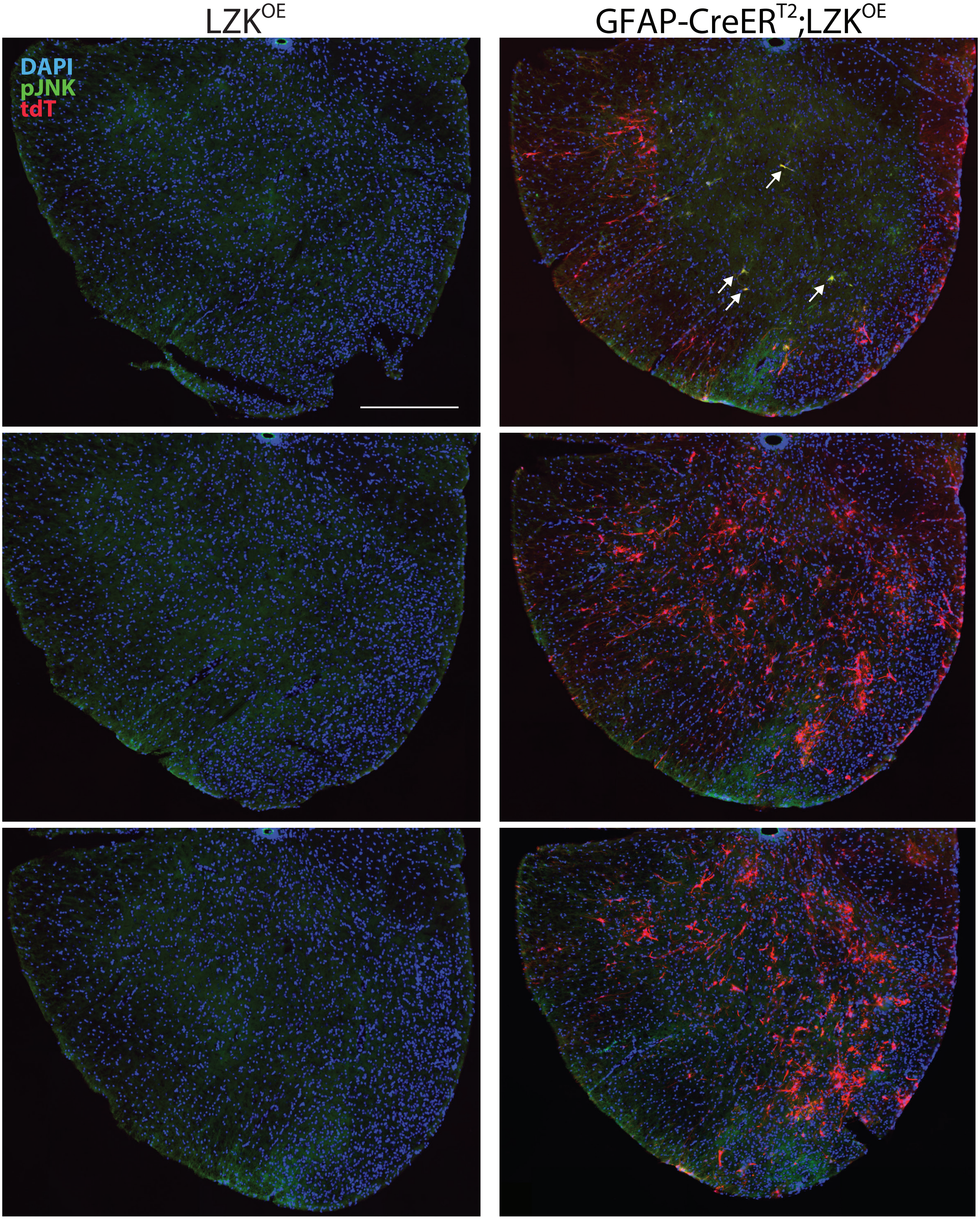

Figure S4

Figure S4. Infrequent detection of JNK activation in adult astrocytes overexpressing LZK without injury. Related to Figure 5. Representative images of DAPI, pJNK, and tdTomato (tdT) immunofluorescence co-labeling in uninjured spinal cords of control LZK^{OE} and GFAP-$CreER^{T2}$;LZK^{OE} mice 3 weeks after the last tamoxifen treatment. Representative sections from 3 control and 3 astrocytic LZK-overexpressing mice are shown (one image per mouse). JNK activation (as assessed by pJNK immunoreactivity) was observed in astrocytes overexpressing LZK-tdT in only one GFAP-$CreER^{T2}$; LZK^{OE} mouse (arrows). Scale bar = 500 µm. Figures are composites of smaller images.

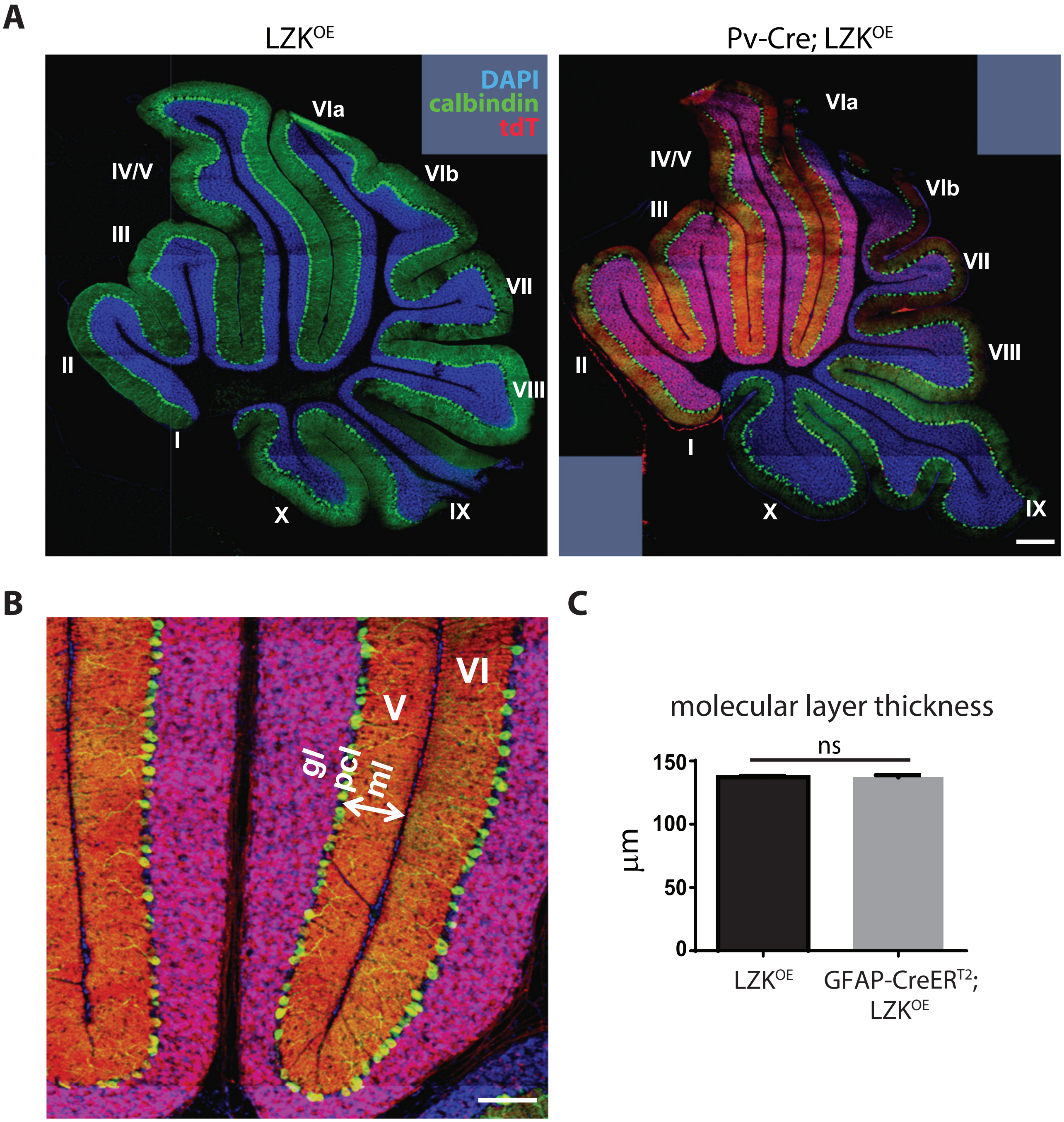

Figure S5. Absence of non-physiological effects of LZK overexpression in parvalbumin (Pv)-expressing neurons. Related to Figure 5. (A) Representative mid-sagittal cerebellar sections from control LZK^{OE} and Pv-Cre;LZK^{OE} mice, at post-natal day 21, co-labeled with Calbindin (Purkinje cell marker), DAPI, and tdTomato. Cerebellar lobules are labeled from I-X. Scale bars = 300 µm. Transgene expression is stronger in Lobules I-V of the cerebellum anterior lobe and lobule VIa of the posterior lobe compared to the other lobules (Hippenmeyer et al, 2005), as shown by tdTomato expression. (B) Representative image showing how molecular layer thickness is measured within the primary fissure. Scale bar = 100 µm. (C) Quantification of molecular layer thickness. Molecular layer (ml); Purkinje cell layer (pcl); granular layer (gl); ns, not significant. Error bar, SEM. Figures are composites of smaller microscopy images.

SUPPLEMENTAL EXPERIMENTAL PROCEDURES

BrdU injections. BrdU (Sigma, B5002) treatment was injected intraperitoneally at 100mg/kg (in 0.007N NaOH and saline) once daily on days 2 through 5 after spinal cord injury.

General histology. Terminal anesthesia was carried out by intraperitoneal injection of pentobarbital sodium or isoflurane overdose. Mice were perfused transcardially with 4% paraformaldehyde, in which dissected brains and spinal cords were further post-fixed overnight at 4°C. Tissues were then cryoprotected in 30% sucrose overnight at 4°C and embedded in O.C.T. compound (Fisher HealthCare) on dry ice. Embedded tissues were sectioned at 20 µm thickness by cryostat (Leica), collected in PBS with 0.01% sodium azide, and further processed for histological examination (see below for details on staining, antibodies and quantification).

Fluorescent immunohistochemistry. Tissue sections were stained as free floating sections. They were first washed twice (wash buffer: 0.2% triton, 1X PBS), at 10 min each. They were then blocked and permeabilized (0.4% triton, 5% sera matching the species of the secondary antibodies, 1X PBS) for 1 hr at room temperature. Incubation in antibody solution (0.2% triton, 1% matching sera, 0.01% sodium azide, 1X PBS) containing desired primary antibody (see concentrations below) was carried out at room temperature overnight. Sections were washed three times, 10 min each, followed by secondary antibody staining for 2 hr at room temperature (antibody solution with desired secondary antibodies used at 1:500). Sections were washed three times, incubated with DAPI (1 µg/ml, 1X PBS) for 10 min, followed by mounting onto glass slides (Fisher Scientific) and cover slipping (Fisher Scientific) with Fluoromount-G (Southern Biotech). For BrdU staining, spinal cords were first incubated in 2N HCl at 37°C for 30 min, rinsed in 0.1M borate buffer at room temperature twice (10 min each), then washed in PBS twice (10 min each). Sections were then subjected to general staining protocol described above with anti-BrdU and anti-GFAP antibodies. For pSTAT3 staining that requires antigen retrieval, spinal cord sections were pre-treated with 1% NaOH for 20 min at room temperature, followed by 3 washes with PBS (10 min each), then incubated with 0.3% glycine in PBS for 10 min, rinsed with PBS (3 times, 10 min each), and finally treated with 0.03% sodium dodecyl sulfate (SDS) in PBS for 10 minutes. After 3 additional washes, sections were blocked in 5% Normal Horse Serum (NHS) in 0.2% Triton X-100 in PBS (PBS-TX) for 1 hr at room temperature, and then incubated with anti-pSTAT3 antibody for overnight at room temperature. The next day, sections were washed 3 times, 10 min per wash in PBS-TX and then incubated in biotinylated anti-rabbit (1:250, in PBS) for 2 hr at room temperature. After 3 washes (10 min each), sections were incubated with ABC solution (in 0.1% Tween-20 1X PBS, Vector Laboratories) over night at 4°C. On the third day, sections were washed with PBS (4 times, 30 min each) then with TSA (Alexa Fluor 488, 1:200 in PBS, Perkin Elmer) for 10 min. After 3 additional washes in PBS, sections were stained for GFAP and tdTomato as described above.

Antibodies. Commercially available antibodies used in this study were: LZK (1:500, rabbit, R06696; Sigma-Aldrich), vimentin (1:500, chicken, ab24525, Abcam), SOX9 (1:500, goat, AF3075, R&D Systems), Ki67 (1:500, rabbit, RM-9106, Thermo Fisher Scientific), GFAP (1:500, rabbit, Z0334, Dako), GFAP (1:500, rat, 130300, Life Technologies), pSTAT3-Tyr705 (1:100, rabbit, 9145, Cell Signaling), tdTomato (1:500, goat, AB8181-200, SICGEN), phospho-JNK(Thr183/Tyr185) (1:300, rabbit, 4668, Cell Signaling), calbindin (1:500, rabbit, 13176, Cell Signaling), BrdU (1:500, rat, ab6326, abcam), Alexa Fluor-tagged secondary antibodies used were Alexa 488, Alexa 546 and Alexa 647 (Thermo Fisher Scientific).

Microscopy and quantification. Stained tissue sections were photographed using an upright epifluorescence microscopy (Zeiss Axio Scan.Z1 and Zeiss Axio Imager M1). After image acquisition, immunofluorescence signal intensity, lesion size, and cell count were determined using the image analysis software ImageJ. For analyses on uninjured brain and spinal cord, 3 mice per genotype, and 3 sections per mouse comparable across animals were used. GFAP immunofluorescence intensity in these animals was quantified as follows: in each coronal brain section, four sampling frames (each of area 440,000 μm^2) were placed within the region of cerebral cortex. In the spinal cord, nine sampling frames or zones (each of length 250 µm and covering the entire width of the cord) were used per section. For SOX9 immunofluorescence intensity quantification, 50 sampling frames (each of area 87 μm^2 surrounding a single $SOX9^+$ nucleus) were placed within the region of interest per section (either the cerebral cortex or gray matter of the spinal cord). For evaluation of signal intensity, integrated density was averaged after subtraction of background signal, and unpaired parametric t-test was used to calculate statistical significance in difference between two groups (GraphPad Prism software). For cell counts, 3 sampling frames of known area were placed within the region of interest per section. All cells positive for signal of interest within each frame was counted (200-600 cells per frame) and normalized to area. Unpaired parametric t-test was used for statistical

evaluation between two groups. To quantify GFAP immunofluorescence intensity in injured spinal cords, 2-3 sections containing the lesion site spanning the entire width of the cord were examined per mouse (see figure legends for group size of spinal cord injury experiments). Nine zones as described above were placed on each section, with the first on the lesion border and the rest placed sequentially away from the injury site and immediately adjacent to each other. After subtraction of background signal, average integrated density for each zone was individually calculated; multiple t-test (paired analysis per zone) was used for statistical evaluation. Vimentin intensity in the injured spinal cord was measured similarly, with only the first zone within 250 µm of the lesion border applied for quantification. Spinal cord injury size was quantified by tracing $GFAP^+$ lesion border in all lesion-containing sections in a set of step-serial horizontal sections (section thickness of 20 µm, staining every 6th section). Lesion area was averaged and unpaired parametric t-test was used to assess statistical significance between two groups. For $pSTAT3^+$ cell counts, the total number of pSTAT3/DAPI co-stained nuclei was quantified at 2 different distances (0-500 µm from the injury site and 1.5-2.0 mm away from the injury) and normalized to the surface of the section measured. 2 sections containing the lesion site spanning the entire width of the cord were examined per mouse (see figure legends for group size). Unpaired parametric t-test was used for statistical evaluation between two groups. For $pSTAT3^+GFAP^+$ cell counts, the number of $pSTAT3^+$ cells overlapping with or tightly encased by $GFAP^+$ astrocytic processes were quantified within a sample region of 200 µm radius. Number of cells were normalized to the area quantified. Two histological sections per mouse were used to generate average cell count per mouse. N = 2 per genotype with no injury; N = 3 per genotype with injury. Unpaired parametric t-test was used for statistical evaluation between two genotypes per condition. For astrocyte proliferation quantification, either the total number of $Ki67^+SOX9^+$ co-labeled nuclei, or the total number of $BrdU^+$ nuclei tightly encased by/overlapping with $GFAP^+$ astrocytic processes within a region spanning the entire width of the spinal cord and within 250 µm of the lesion border (excluding ependymal cells lining the central canal) was counted and normalized to area. Two sections per mouse were quantified. Unpaired parametric t-test was used for statistical evaluation between two genotypes. Thickness of the cerebellar molecular layer was measured within the primary fissure between lobules V and VI on sagittal sections. 2-3 midline sections per mouse, three mice per genotype were used for quantification. Unpaired t-test was used for statistical evaluation between two genotypes. To quantify the number of LZK^+GFAP^+ cells in the uninjured and injured spinal cords, such cells in a sample region of grey matter approximately $0.2mm^2$ in size were counted and normalized to the size of the area quantified. Two histological sections per animal were used to generate average cell count per animal. N = 3 per condition/genotype. Unpaired parametric t-test was used for statistical evaluation between two groups.
